# Grain-maturing temperature induces seed epi-memory via DNA methylation for subsequent development in rice (*Oryza sativa* L.)

**DOI:** 10.1093/plphys/kiag219

**Published:** 2026-04-21

**Authors:** Chetphilin Suriyasak, Yui Oyama, Ryusuke Kawaguchi, Ryo Matsumoto, Yuta Sawada, Wun-Jin Chen, Hue Thi Nong, Norimitsu Hamaoka, Yushi Ishibashi

**Affiliations:** Faculty of Agriculture, Kyushu University, Fukuoka 819-0395, Japan; Graduate School of Bioresource and Bioenvironmental Sciences, Kyushu University, Fukuoka 819-0395, Japan; Graduate School of Bioresource and Bioenvironmental Sciences, Kyushu University, Fukuoka 819-0395, Japan; Graduate School of Bioresource and Bioenvironmental Sciences, Kyushu University, Fukuoka 819-0395, Japan; Graduate School of Bioresource and Bioenvironmental Sciences, Kyushu University, Fukuoka 819-0395, Japan; Graduate School of Bioresource and Bioenvironmental Sciences, Kyushu University, Fukuoka 819-0395, Japan; Graduate School of Bioresource and Bioenvironmental Sciences, Kyushu University, Fukuoka 819-0395, Japan; Graduate School of Bioresource and Bioenvironmental Sciences, Kyushu University, Fukuoka 819-0395, Japan; Faculty of Biotechnology, Vietnam National University of Agriculture, Hanoi, 131000, Vietnam; Tropical Crop and Environment Section, Institute of Tropical Agriculture, Kyushu University, Fukuoka 819-0395, Japan; Faculty of Agriculture, Kyushu University, Fukuoka 819-0395, Japan; Graduate School of Bioresource and Bioenvironmental Sciences, Kyushu University, Fukuoka 819-0395, Japan

## Abstract

High temperature during grain filling diminishes the yield and quality of rice (*Oryza sativa* L.), but whether and how it affects development and agronomic traits in the subsequent plants remain unclear. In this study, we dissected the DNA methylome of seeds that developed under heat stress (heat-treated during development seeds, HDS) or control seeds (CS), and the transcriptomes of the plants derived from these seeds. Our methylome analysis identified 457 differentially methylated regions in HDS, mostly at the promoter regions of protein-coding genes and transposons. Transcriptome analysis detected 2,824 differentially expressed genes (DEGs) between plants grown from HDS or CS. HDS-derived subsequent plants were shorter, produced more tillers, had greater stomatal density, flowered earlier, and exhibited earlier diurnal floret opening than CS-derived plants. We linked DEGs to these phenotypic changes: *OsSLB1* and *OsYODA1* were downregulated, promoting abundant tillering and high stomatal density, respectively; *OsHd1* and *OsAOC1* were upregulated, leading to early flowering and early diurnal floret opening, respectively. In HDS, the *OsSLB1* and *OsYODA1* promoters were hypermethylated, but the *OsHd1* and *OsAOC1* promoters were hypomethylated, relative to CS. Notably, these seed methylation differences remained in their derived subsequent plants. Finally, yield was 9.5% higher for HDS-derived plants than for CS-derived plants when cultivated in the field. Our findings suggest the importance of seed epigenetic memory, mediated by DNA methylation, for the development of preferred agronomic traits.

## Teaser

Alterations in seed DNA methylation due to grain-maturing temperature induce favorable adaptive traits in rice.

## Introduction

Recent changes in climate due to global warming are having profound effects on agricultural production, which is essential for food and economic security worldwide. As global temperatures rise, the yields of important crops such as maize (*Zea mays*), soybean (*Glycine max*), and cotton (*Gossypium* sp.) have suffered marked drops as a result of plants experiencing high temperatures; these effects are predicted to worsen before the end of the century, with predicted yield losses of up to 46% (Schlenker and Roberts 2009). One estimate indicates that drought stress and extreme heat stress from 1964 to 2007 led to global declines of 9% to 10% in the production of cereals, including maize, wheat (*Triticum aestivum*), and rice (*Oryza sativa*) (Lesk et al. 2016). As the world population keeps growing, it is crucial to mitigate the effects of high temperatures on crop yields (Schauberger et al. 2016; Shew et al. 2020). Rice, an important crop that feeds about half of the world's population (Seck et al. 2012), is severely affected by heat stress: one study predicts that each 1 °C rise in minimum temperature during the growing season will cause a 10% decline in grain yield (Peng et al. 2004). In *japonica* rice cultivars, air temperatures above 26 °C during the grain-filling stage lead to chalky grains, resulting in lower grain weight and quality (Yamakawa et al. 2007; Hakata et al. 2012). Our previous studies also show that temperature treatment of 30 °C inside the phytotron significantly causes grain chalkiness occurrence (Funaba et al. 2006; Tanaka et al. 2009; Tanamachi et al. 2016; Suriyasak et al. 2017, 2025). Although many studies have explored the effects of heat stress on yield and quality in rice, whether and how heat stress during the grain-filling stage affects the growth and development of plants derived from these heat-exposed seeds is not yet well understood.

In many plant species, responses to environmental changes are transferred from a plant to its progeny (Whittle et al. 2009; Wang et al. 2016). For example, *Arabidopsis* (*Arabidopsis thaliana*) plants germinated from seeds collected from plants subjected to repeated heat stress treatment produce more flowers and seeds under high-temperature conditions (Whittle et al. 2009) and have an altered flowering time (Groot et al. 2017). This so-called epigenetic memory or transgenerational memory has been reported in dicot and monocot species, including *Arabidopsis* (Whittle et al. 2009), peanut (*Arachis hypogaea*) (Racette et al. 2020), wheat (Wang et al. 2016), and rice (Zheng et al. 2017), when exposed to various abiotic stresses, representing a mechanism that promotes the development and acclimation of the progeny in response to these stressful conditions (He and Li 2018; Perrella et al. 2022). Epigenetic marks such as histone modifications and DNA methylation help transfer this memory from the parents to their progeny (Kou et al. 2011; Zheng et al. 2017; Liu et al. 2019).

DNA methylation is responsible for the long-term stress memory that shapes development and environmental responses via transcriptional regulation (Crisp et al. 2016; Lang et al. 2017). In plants, DNA methylation of cytosines occurs in 3 contexts, namely CG, CHG, and CHH (H stands for A, T, or C), to modulate gene expression and suppress transposable element (TE) activity (Law and Jacobsen 2010; Xu et al. 2020). When rice is exposed to drought stress over several generations, DNA methylation patterns change in a nonrandom manner between accumulative generations to promote subsequent development and stress tolerance (Zheng et al. 2017). In our previous studies, we demonstrated that heat stress during grain-filling alters DNA methylation levels at the promoters of germination-related genes in mature seeds, leading to transcriptional changes that delay or promote subsequent germination in rice and barley (*Hordeum vulgare*), respectively (Suriyasak et al. 2020; Sakai et al. 2022). Similarly, changes in DNA methylation in seeds at the promoters of starch metabolism genes in response to heat stress during grain-filling affect the transcriptional output of these genes, resulting in an effective acquired thermotolerance for heat-exposed seeds and leading to better grain quality of the subsequent plants (Suriyasak et al. 2025). These findings underscore the importance of DNA methylation in seeds for shaping transcription during seed germination and plant development, which might influence the subsequent growth and modulate agronomic traits. Therefore, this study aimed to explore how heat stress during grain-filling alters the global methylome landscape in seeds and how these changes regulate subsequent development in rice, which might offer a promising and sustainable means for improving rice production in the face of worsening global warming.

## Results

### Alterations of the DNA methylation landscape in seeds developed under heat stress during grain filling

**Figure 1:**
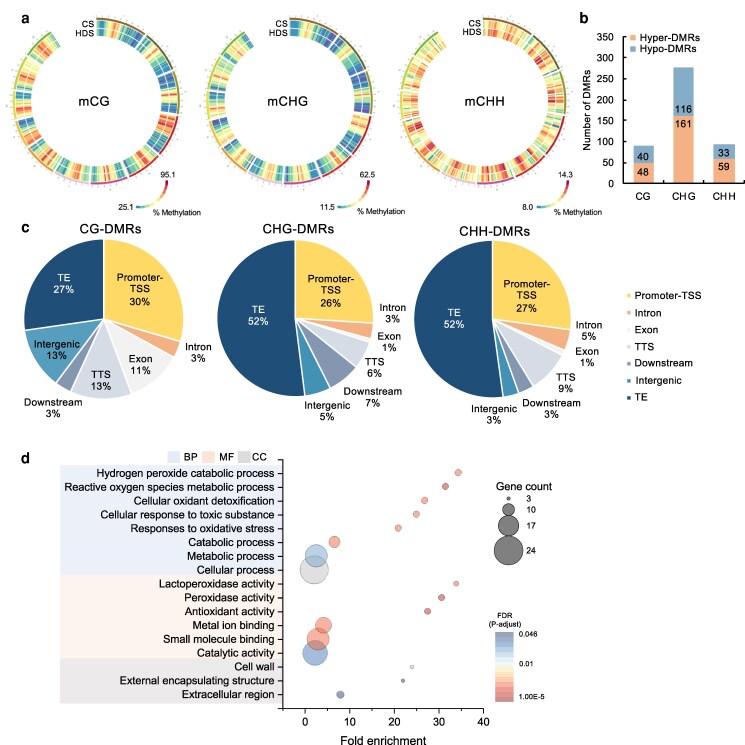
The global methylome landscape of seeds that were heat-treated during grain-filling differs from that of CS. (a) Circos plots showing the average total methylation levels in each methylation context (CG, CHG, CHH) in 1-Mb nonoverlapping windows in CS and heat-treated during development seeds (HDS). (b) Number of hyperdifferentially methylated regions (hyper-DMRs) and hypo-DMRs in HDS relative to CS (Total DMR = 457; hyper-DMR = 268, hypo-DMR = 189) and (c) distribution of DMRs in different annotated genomic regions in each methylation context. TE, transposable element; TSS, transcription start site; TTS, transcription termination site. (d) Enriched GO terms among all identified seed DMRs in HDS compared with CS (BP, biological process; MF, molecular function; CC, cellular component; FDR, false discovery rate).

We grew plants from the rice cultivar “Nipponbare” under natural conditions until ovule fertilization before dividing the plants into 2 sets. We exposed each set to a different temperature regime: either control (25 °C) or heat (30 °C), at the grain-filling stage until they were fully mature. We harvested the resulting seeds, which we designated CS for control seeds and heat-treated during development seeds (HDS) for heat-treated developed seeds (Figure S1a). To determine how heat stress during grain-filling affects DNA methylation in matured seeds, we identified all methylated cytosines at single-base resolution by whole-genome bisulfite sequencing (WGBS) to generate global methylome landscapes for CS and HDS. Analysis of the WGBS data showed methylation of 56.6% of cytosines in the CG context, 27.0% in the CHG context, and 14.1% in the CHH context in CS. In HDS, 1% to 1.5% more cytosines were methylated compared with CS across all cytosine contexts, reaching 57.8% (CG), 28.5% (CHG), and 15.5% (CHH) (Table S1). The average methylation levels of cytosines were plotted in 1-Mb windows for each of the 3 methylation contexts along all chromosomes (Fig. 1a). We then searched for differentially methylated regions (DMRs) in HDS relative to CS, leading to 457 DMRs, with 270 being hypermethylated and 187 hypomethylated (Fig. 1b). These DMRs in all methylation contexts were mostly present within the promoter regions of protein-coding genes upstream of transcription start sites (TSSs) and in TEs (Fig. 1c). Retrotransposons were the most frequent TE type to which DMRs mapped (Figure S2). A gene ontology (GO) term analysis revealed that the total DMRs detected in seeds map to genes involved in redox responses, including hydrogen peroxide catabolism, reactive oxygen species (ROS) metabolism, responses to oxidative stress, and antioxidant activity (Fig. 1d). These results suggest that heat stress during grain-filling alters global DNA methylation levels in seeds.

### Heat stress during grain-filling induces transcriptional changes during subsequent plant development

**Figure 2:**
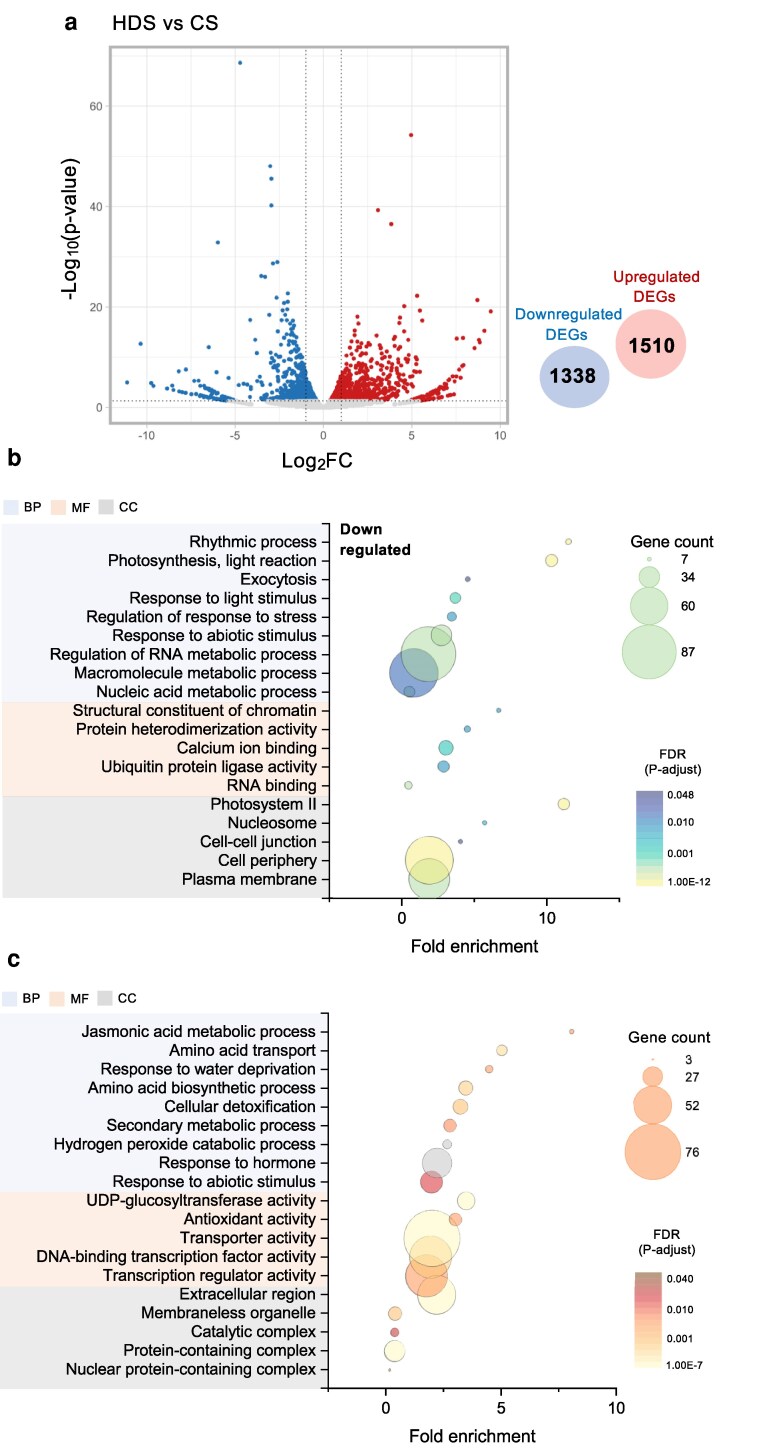
Plants derived from HDS and CS show transcriptional differences under the same growing natural condition. (a) Volcano plot of DEGs identified from RNA-seq in developing leaves during vegetative stages of plants grown from HDS relative to those in plants grown from CS under natural conditions (CS, control seeds; HDS, heat-developed seeds). Enriched GO terms among (b) downregulated DEGs and (c) upregulated DEGs (BP, biological process; MF, molecular function; CC, cellular components; FDR, false discovery rate).

We performed a transcriptome deep sequencing (RNA-seq) analysis of fully developed leaves, collected at the vegetative stage at 55 d after sowing (DAS) from plants derived from CS and HDS grown under natural conditions (Figure S1b). For clarity and brevity, the plants derived from those seeds are referred to hereafter as CS or HDS plants. We identified 2,848 significant differentially expressed genes (DEGs) in the leaves of HDS plants compared with those of CS plants, with 1,510 upregulated and 1,338 downregulated genes (Fig. 2a). A GO term analysis of these DEGs indicated that the downregulated DEGs are involved in many processes, including photosynthesis, being associated with the terms “light reaction” and “photosystem II,” in addition to the term “nucleic acid metabolism” (Fig. 2b). Upregulated DEGs showed an enrichment for the terms “response to phytohormones” and “response to water deprivation,” as well as redox-related GO terms including “hydrogen peroxide catabolism,” “detoxification,” and “antioxidant activity” (Fig. 2c). Notably, the GO term “response to abiotic stimulus” was shared by downregulated and upregulated DEGs. These results suggest that HDS plants show significant differences in transcript levels during plant development compared with CS plants, even when grown under the same growth conditions devoid of heat stress.

### Heat stress during grain-filling promotes tillering and early heading in the subsequent plants

**Figure 3:**
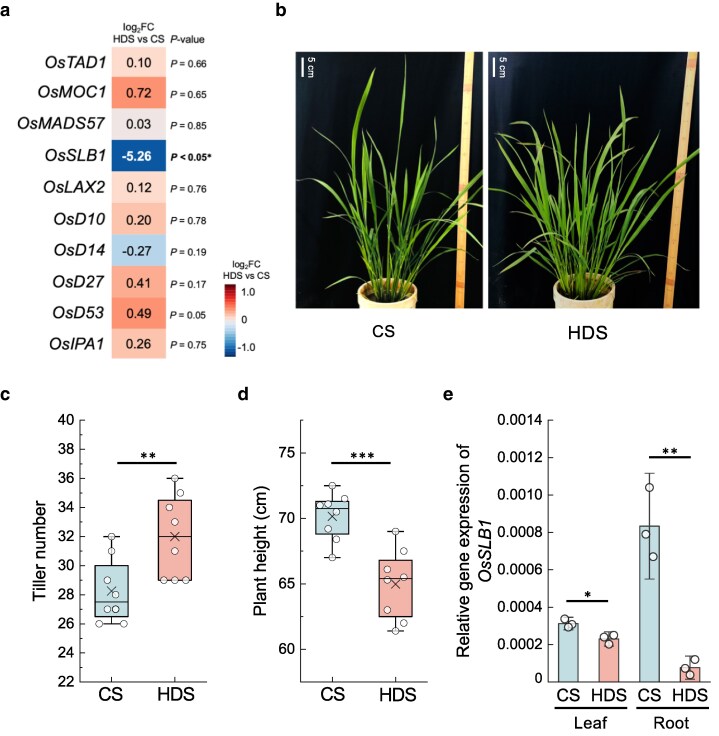
Subsequent plants derived from HDS develop higher tillering phenotype. (a) Heatmap representation of expression for genes regulating tillering (*OsTAD1*, Os03g0123300; *OsMOC1*, Os06g0610350; *OsMADS57*, Os02g0731200; *OsSLB1*, Os01g0700900; *OsLAX2*, Os04g0396500; *OsD10*, Os01g0746400; *OsD14*, Os03g0203200; *OsD27*, Os11g0587000; *OsD53*, Os11g0104300; *OsIPA1*, Os08g0509600). Values are based on RNA-seq data. (b) Representative photographs of plants derived from HDS and CS at tillering stage under natural conditions (CS, control seeds; HDS, heat-developed seeds). (c) Tiller number and (d) plant height (*n* = 8) of CS and HDS plants at the tillering stage. In boxplots, the upper and lower bounds represent the 75th and 25th percentiles, respectively. The medians are shown as lines within the box, with the cross marks representing the averages. Whiskers represent SD values. (e) Relative *OsSLB1* expression levels via RT-qPCR in leaves and roots of CS and HDS plants at the tillering stage (*n* = 3) in 2019. Error bars represent SD values. Asterisks indicate significant differences, as determined by Student's *t*-test (*, *P* < 0.05; **, *P* < 0.01; and ***, *P* < 0.001; N.S., not significant).

We looked for DEGs with known roles in shaping agronomic traits. Here, *STRIGOLACTONE BIOSYNTHESIS1* (*OsSLB1*), an ortholog of *Arabidopsis MORE AXILLARY GROWTH1* (*MAX1*) that controls shoot lateral branching (Cardoso et al. 2014), was of particular interest due to its role in suppressing tillering while inducing plant height in rice (Wang and Li 2011). *OsSLB1* was among the downregulated DEGs and its expression was significantly lower in HDS plants, which other tillering-regulated genes were not differentially expressed (Fig. 3a). An observation of phenotypes in CS and HDS plants confirmed that HDS plants develop more tillers with reduced height compared with CS plants (Fig. 3c and d;  Figure S3a and b). Since strigolactone is biosynthesized mainly in roots and moves acropetally to shoots, where it suppresses tillering while inducing stem growth (Wang and Li 2011; Hussien et al. 2014; Liu et al. 2020), we confirmed the expression levels of *OsSLB1* via reverse-transcription quantitative PCR (RT-qPCR) in leaves and roots at tillering stage. *OsSLB1* expression was significantly lower by about 25% and 90% in leaves and roots, respectively, of HDS plants compared with that in CS plants (Fig. 3e), suggesting a role in enhancing tillering in HDS plants.

**Figure 4:**
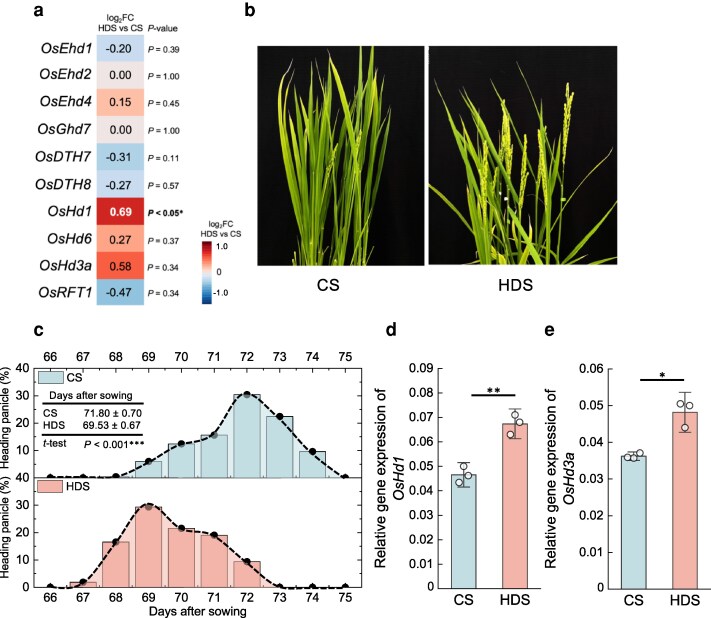
Subsequent plants derived from HDS show early heading phenotype. (a) Heatmap representation of expression for genes regulating heading (*OsEhd1*, Os10g0463400; *OsEhd2*, Os10g0419200, *OsEhd4*, Os03g0112700; *OsGhd7*, Os07g0261200; *OsDTH7*, Os07g0695100; *OsDTH8*, Os08g0174500; *OsHd1*, Os06g0275000; *OsHd6*, Os03g0762000; *OsHd3a*, Os06g0157700; *OsRFT1*, Os06g0157500). Values are based on RNA-seq data. (b) Heading phenotype and (c) distribution of panicle heading on each date for CS and HDS plants (CS = 250 plants, HDS = 320 plants) observed in 2020 (CS, control seeds; HDS, heat-developed seeds). The average heading dates ± standard deviation of all pots (CS = 25 pots, HDS = 32 pots) are shown on top. (d) Relative *OsHd1* and (e) *OsHd3a* expression levels via RT-qPCR in the leaves of plants derived from CS and HDS at 55 DAS (*n* = 3). Values are means ± SD. Asterisks indicate significant differences, as determined by Student's *t*-test (*P < 0.05; **P < 0.01; and ***P < 0.001).

Aside from *OsSLB1* related to tillering, we also noticed *Heading date1* (*OsHd1*), an ortholog of *Arabidopsis CONSTANS* that plays an important role in flowering time regulation (Hayama et al. 2003; Ishikawa et al. 2011; Nemoto et al. 2016) among the upregulated DEGs. *OsHd1* was the only heading-regulated gene whose expression was significantly higher in the leaves of HDS plants than in those of CS plants (Fig. 4a), suggesting a possible role in heading timing in HDS plants. In agreement with the *OsHd1* expression pattern, HDS plants reached the heading stage about 2 d earlier than CS plants when grown under the same normal growth conditions (natural short-day [SD] condition), a difference that was significant (Fig. 4b and c). We validated the higher expression levels of *OsHd1* in the leaves of HDS plants via RT-qPCR, detecting a 44% higher expression for *OsHd1* in HDS than in CS plants (Fig. 4d). We also detected upregulation of the downstream OsHd1 target gene *OsHd3a*, an ortholog of *Arabidopsis FLOWERING LOCUS T* (*FT*) (Fig. 4e). In addition, as OsHd1 induces heading under SD conditions but represses heading under long-day (LD) conditions (Ishikawa et al. 2011; Liu et al. 2014; Nemoto et al. 2016), we tested the heading behavior of CS and HDS plants when grown under LD conditions. Exposing seeds to heat stress during their grain-filling stage significantly delayed heading of the subsequent plants by over 1 d; *OsHd1* expression remained significantly higher in HDS plants relative to CS plants when grown under LD conditions (Figure S4a to c).

### Heat stress during grain-filling raises stomatal density and promotes early floret opening in the subsequent plants

**Figure 5:**
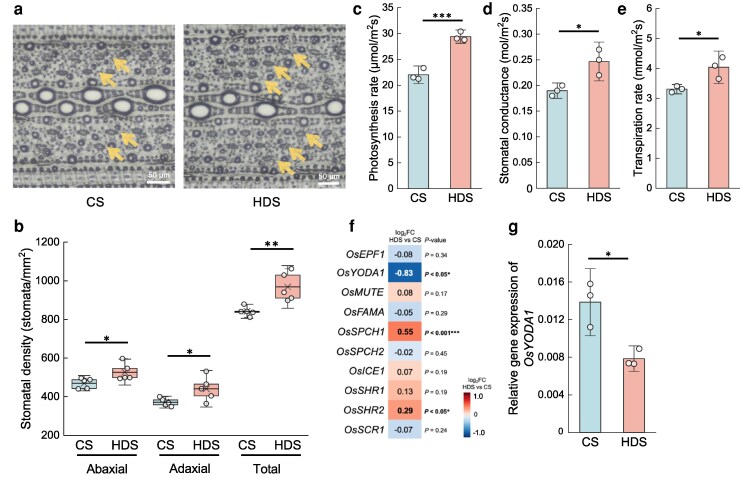
Subsequent plants derived from HDS have a higher stomatal density and higher photosynthetic performances. (a) Imprints of stomata from the abaxial surface of leaves from CS and HDS plants (CS, control seeds; HDS, heat-developed seeds). Arrows indicate stomata. (b) Stomatal density on the leaves of CS and HDS plants (*n* = 5) observed in 2022. (c) Photosynthetic rates; (d) stomatal conductance and (e) transpiration rates of measured in CS and HDS leaves at 62 DAS observed in 2022 (*n* = 3). In boxplots, the upper and lower bounds represent the 75th and 25th percentiles, respectively. The medians are shown as lines within the box, with the cross marks representing the averages. Whiskers represent SD values. (f) Heatmap representation of expression levels for genes related to stomatal development and (g) *OsYODA1*, in developing seedlings (*n* = 3) as determined by RT-qPCR. Error bars represent SD values. Asterisks indicate significant differences, as determined by Student's *t*-test (*P < 0.05; **P < 0.01; and ***P < 0.001).

HDS plants exhibited phenotypic changes including greater tillering and early heading, which were reflected by the differential expression of several genes in leaves during the vegetative stage. We also observed other phenotypic changes, as HDS plants had a significantly greater stomatal density on the epidermis of their leaves compared with that of CS plants (Fig. 5a and b). The higher stomatal density was associated with a higher photosynthetic rate, greater stomatal conductance, and a higher transpiration rate in HDS plants (Fig. 5c to e). Biomass accumulation also improved, as HDS seedlings produced 41% more biomass than their CS counterparts when grown under ambient CO_2_ levels (400 ppm), reaching 67% more biomass under elevated CO_2_ conditions (800 ppm) (Figure S5a and b). In addition, leaves of HDS plants were cooler, indicative of higher transpiration, than CS plants when exposed to 35 °C heat stress (Figure S6). Since the RNA-seq analysis was based on mature leaves, we turned to RT-qPCR to assess the expression levels of genes related to stomatal development in developing young HDS and CS seedlings (Fig. 5f;  Figure S7a to i). The expression of *OsYODA1*, encoding a mitogen-activated protein kinase kinase kinase, a negative regulator of the stomatal lineage and stomata differentiation (Bertoni 2009; Abrash et al. 2018), was 60% lower in HDS seedlings relative to that in CS seedlings (Fig. 5f and g). Expression levels of the downstream genes of OsYODA1, *SPEECHLESS1* (*OsSPCH1*), and *SHOOTROOT2* (*OsSHR2*) were higher in HDS seedlings (Fig. 5f), which may help to explain the higher stomatal density phenotype of HDS plants.

**Figure 6:**
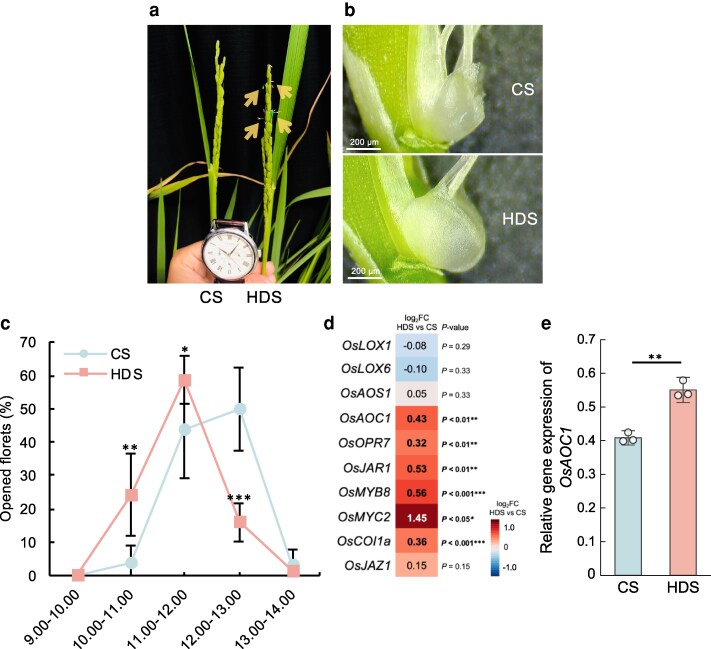
Subsequent plants derived from HDS show an early diurnal floret opening phenotype. (a) Representative photographs (arrows indicate open florets) and (b) lodicules of CS and HDS plants during floret opening (CS, control seeds; HDS, heat-developed seeds). (c) Distribution of open florets at different time points during the daytime (*n* = 6) observed in 2018. (d) Heatmap representation of expression levels for genes related to JA biosynthesis and signaling and (e) *OsAOC1* expression in CS and HDS lodicules at 10:00 AM (*n* = 3), as determined by RT-qPCR analysis. Error bars represent SD values. Asterisks indicate significant differences, as determined by Student's *t*-test (*P < 0.05; **P < 0.01; and ***P < 0.001).

We also determined that, in addition to early heading, the florets of HDS plants opened significantly earlier during the day than those of CS plants. Indeed, the florets of HDS plants started to open with lodicule expansion around 10:00 AM and reached a peak in opening around 11:00 to 12:00, or about 1 h earlier than those of CS plants (Fig. 6a to c). We checked the transcript levels of genes involved in the jasmonic acid (JA) signaling pathway, which has been proposed to play important roles in diurnal floret opening in rice, (Gou et al. 2024; Wang et al. 2024) using RT-qPCR in CS and HDS lodicules (Fig. 6d;  Figure S8a to i). *ALLENE OXIDE CYCLASE1* (*OsAOC1*), encoding an enzyme catalyzing the formation of the JA precursor 2-oxo-phytodienoic acid, was significantly upregulated in HDS lodicules, as were the downstream biosynthesis and signaling genes *12-OXOPHYTODIENOATE REDUCTASE7* (*OsOPR7*), *JASMONYL-L-ISOLEUCINE SYNTHASE1* (*OsJAR1*), *OsMYB8*, *OsMYC2*, and *CORONATINE INSENSITIVE 1a* (*OsCOI1a*) (Fig. 6d and e). Together, these results suggest that heat stress during grain-filling significantly alters the transcript levels of several genes, leading to phenotypic changes including greater tillering, higher stomatal density, early heading, and early diurnal floret opening in HDS plants. Importantly, we confirmed all these phenotypic changes in other experimental years (Figure S9a to i).

### Alterations of DNA methylation levels in heat-developed seeds remain in developing organs of the subsequent plants

**Figure 7:**
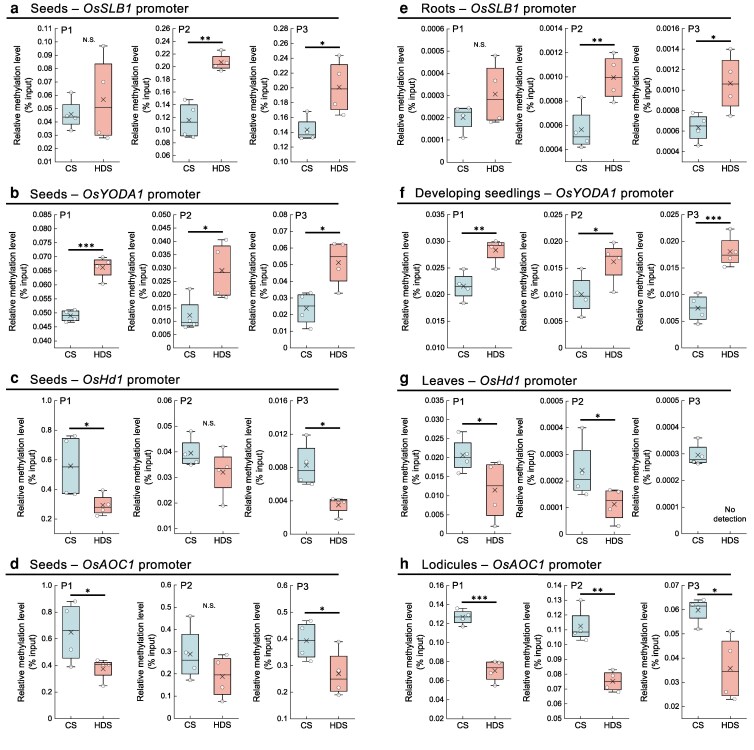
Relative methylation levels in CS and HDS at the promoters of genes related to the observed phenotypic changes are retained in progeny plants. Relative methylation levels at the promoter regions of (a) *OsSLB1*, (b), *OsYODA1*, (c), *OsHd1*, and (d) *OsAOC1* in CS and HDS (CS, control seeds; HDS, heat-developed seeds). (e) Relative methylation levels at the *OsSLB1* promoter regions in roots at the tillering stage. (f) Relative methylation levels at the *OsYODA1* promoter regions in developing seedlings. (g) Relative methylation levels at the *OsHd1* promoter in leaves. (h) Relative methylation levels at the *OsAOC1* promoter in lodicules. P1–P3 show different specific MeDIP-qPCR amplicons covering the promoter regions. Data are shown as boxplots from 4 biological replicates, each shown as individual circles. In boxplots, the upper and lower bounds represent the 75th and 25th percentiles, respectively. The medians are shown as lines within the box, with the cross marks representing the averages. Whiskers represent SD values. Asterisks indicate significant differences, as determined by a Student's *t*-test (*P < 0.05; **P < 0.01; and ***P < 0.001; N.S., not significant).

Our previous studies have shown that alterations of seed DNA methylation in response to heat stress during grain-filling affect transcript changes in the subsequent development (Suriyasak et al. 2020, 2025; Sakai et al. 2022). Here, to elucidate the roles of seed DNA methylation on subsequent development via regulating the target genes listed above, we performed methylated DNA immunoprecipitation quantitative PCR (MeDIP-qPCR) to assess the loci-specific quantitative methylation levels of the *OsSLB1*, *OsYODA1*, *OsHd1*, and *OsAOC1* promoters in harvested seeds and in developing organs of CS and HDS plants. The *OsSLB1* and *OsYODA1* promoter regions were significantly more methylated in HDS relative to CS (Fig. 7a and b). Importantly, these promoters remained more methylated in developing HDS roots (*OsSLB1*) and seedlings (*OsYODA1*) (Fig. 7e and f); both genes were downregulated in each organ of HDS plants (Figs. 3e and 5g). By contrast, the *OsHd1* and *OsAOC1* promoter regions were significantly less methylated in HDS than in CS (Fig. 7c and d), as well as in leaves and in lodicules of HDS plants (Fig. 7g and h); both genes were upregulated in each organ of HDS plants (Figs. 4d and 6e). Therefore, the DNA methylation status of the *OsSLB1*, *OsYODA1*, *OsHd1*, and *OsAOC1* promoter regions observed in HDS is maintained in developing organs of HDS plants, which may regulate transcript levels and lead to the developmental changes observed in HDS plants.

### Heat stress during grain-filling improves yield in subsequent HDS plants

Last, we conducted field experiments to explore the consequences of heat exposure during grain filling on subsequent agronomic traits. Accordingly, we transplanted CS and HDS seedlings under natural field conditions and measured the final yields of CS and HDS plants. In a field experiment conducted in 2020 and 2024, the total yields of HDS plants were 9.5% and 9.4% higher than that of CS plants in 2020 and 2024, respectively, and were associated with a significantly greater panicle number, despite no change in other yield components and harvest index (Table 1). In addition, we observed similar results in individual HDS plants grown in pots, with significantly higher total yields of 14.5% and 38.7% in 2019 and 2023, respectively, due to increase in panicle numbers (Table S2). Therefore, these results suggest that heat stress during grain-filling leads to higher yields of the subsequent plants growing at natural condition.

**Table 1:** Yield components of field experiments for plants grown from CS and heat-treated developed seeds (HDS) in 2020 and 2024 under natural field conditions.

Field experiment in 2020	Panicle number	Spikelet/panicle	Grain filling (%)	1,000-grain weight (g)	Total yield (g/m^2^)	Harvest index
**CS**	331 ± 9.53	92.27 ± 3.42	93.22 ± 2.01	19.55 ± 0.10	570.54 ± 19.58	0.45 ± 0.01
**HDS**	356 ± 12.16	92.51 ± 7.64	91.22 ± 3.08	19.86 ± 0.39	625.28 ± 31.65	0.47 ± 0.01
***P*-value (*n* = 3)**	*P* = 0.025*	*P* = 0.407	*P* = 0.214	*P* = 0.392	*P* = 0.037*	*P* = 0.062

Field experiment in 2024	Panicle number	Spikelet/panicle	Grain filling (%)	1000-grain weight (g)	Total yield (g/m^2^)	Harvest index
**CS**	502.33 ± 16.01	75.76 ± 2.05	81.35 ± 3.56	19.09 ± 0.76	633.24 ± 34.70	0.45 ± 0.02
**HDS**	530.33 ± 4.61	75.36 ± 3.05	83.16 ± 2.86	19.88 ± 0.45	693.06 ± 29.96	0.48 ± 0.02
***P*-value (*n* = 3)**	*P* = 0.042*	*P* = 0.430	*P* = 0.265	*P* = 0.108	*P* = 0.044*	*P* = 0.104

Values are means ± SD (*n* = 3). Asterisks indicate significant differences, as determined by a Student's *t*-test (*P < 0.05). CS, control seeds; HDS, heat-developed seeds.

## Discussion

Up until recent, although multiple studies have reported changes in the methylome landscape in developing seeds (An et al. 2017; Wang et al. 2017), corresponding datasets for fully mature harvested seeds, especially matured under environmental stress, are limited. Here, we performed a WGBS analysis, which revealed an ∼1% rise in global cytosine methylation levels in seeds that were HDS compared with CS (Table S1), together with more hyper-DMRs than hypo-DMRs (Fig. 1b). Although the mechanism underlying hypermethylation in seeds developed under heat stress is still unclear, a previous study suggested that genome-wide hypermethylation in dry seeds reinforces chromatin packing, which would prevent the induction of gene expression and TE activation prior to seed germination (Kawakatsu et al. 2017). Thus, the possible effects of heat stress during grain-filling on seed chromatin remodeling should be elucidated in the future. In HDS, we detected more CG DMRs mapping to the promoters than we did DMRs in the other 2 contexts, while TEs harbored more non-CG DMRs than CG DMRs (Fig. 1c). Methylation in the promoter of a protein-coding gene is associated with its transcriptional regulation (Zhang et al. 2006). Most of the TEs to which DMRs mapped were retrotransposons (Figure S2), which are known to respond to various stimuli and affect gene expression (Galindo-Gonzalez et al. 2017; Liu et al. 2020). Our previous study showed that heat stress during grain-filling induces ROS production in developing rice grains (Suriyasak et al. 2017); therefore, it might be possible that greater ROS production during grain-filling affects seed ROS homeostasis, reflecting the enrichment of redox- and oxidative stress-related total DMRs in HDS (Fig. 1d).

Our results indicate that rice plants grown from seeds having matured under 30 °C exhibit differences in their transcript levels and in several phenotypes during their development compared with plants from seeds matured under 25 °C (Fig. 2a to c). Plants grown from HDS developed more tillers and were shorter than those grown from CS (Fig. 3b to d;  Figure S3a and b). The plant hormone strigolactone dictates tillering number and height in rice (Liu et al. 2020); in agreement with this notion, the strigolactone biosynthetic gene *OsSLB1* was strongly downregulated in the roots and shoots of HDS plants, likely promoting tillering (Fig. 3e). A previous study reported that *japonica* rice subspecies producing high levels of strigolactone are characterized by a small tiller number, while *indica* subspecies with low strigolactone levels produce many tillers (Cardoso et al. 2014). In addition, overexpression of *OsSLB1* in the *Arabidopsis max1* mutant background rescues the high branching phenotype of this mutant (Cardoso et al. 2014), supporting the roles of strigolactone in branching in both monocots and dicots. Tillering or branching is a crucial agronomic trait (Hussien et al. 2014) that determines final yield at harvest for many important crop plants, including barley (Moral and Moral 1995), spring wheat (Sharma 1995), and soybean (Carpenter and Board 1997). In rice, the number of tillers during plant development significantly correlates with panicle number at harvest, thus determining final yield (Badshah et al. 2014), as observed in HDS plants. In addition, we observed the downregulation of *OsYODA1*, a negative regulator of stomatal development, together with a higher stomatal density in HDS plants (Fig. 5b and g). YODA1 is conserved in dicot and monocot species and is a key factor suppressing the expression of the downstream gene *SPCH* to control stomatal lineage and differentiation (Bergmann and Sack 2007; Bertoni 2009). In *Arabidopsis*, overexpression of *YODA1* blocks stomatal development, while loss of YODA1 activity results in excess production of stomatal clusters (Bergmann et al. 2004; Samakovli et al. 2020). In the monocot plant purple false brome (*Brachypodium distachyon*), the *Bdyda1* mutant also has more stomatal clusters and displays stomatal patterning defects (Abrash et al. 2018). In fact, genetic manipulation to increase stomatal density does not always result in biomass accumulation (Tanaka et al. 2013), since it creates a trade-off between CO_2_ uptake and water transpiration (Buckley et al. 2020). Here, self-manipulated increase in stomatal density of HDS plants did not show any trade-off, since HDS plants accumulated more biomass, which was more apparent under elevated CO_2_ conditions (Figure S5). Stomatal density is positively correlated with CO_2_ uptake in rice through photosynthesis (Xiong et al. 2022), which is consistent with the higher photosynthetic activity measured in HDS plants (Fig. 5c). Taken together, we speculate that the suitable increase in stomatal density of HDS plants facilitates biomass accumulation, which combines with their high-tillering phenotype to contribute to their higher yield at harvest. Of note, a higher stomatal density is also responsible for cooling leaves via evapotranspiration under heat stress (Rathnasamy et al. 2023), as HDS seedlings were more thermotolerant than CS plants when exposed to 35 °C heat stress (Figure S6). Our previous study also showed that heat stress during grain-filling induces subsequent thermotolerance in rice, resulting in higher photosynthesis and transpiration rates, as well as delayed leaf senescence, leading to higher grain quality under heat stress (Suriyasak et al. 2025), possibly through higher stomatal density as reported in this study. Therefore, we suggest that heat stress during grain-filling improves the yield and thermotolerance of the subsequent plants.

The warm or cool climates of different growing areas influence progeny heading date in rice, reflecting the water temperature during vegetative growth (Koumoto et al. 2018). We demonstrated here that heat stress during grain-filling results in an early heading phenotype in HDS plants compared with CS plants under the same growing conditions (Fig. 4b and c). In the plants grown from of HDS and CS, *OsHd1* expression was significantly upregulated in HDS plants, as was *OsHd3a*, a known target of OsHd1 that promotes heading (Fig. 4d and e). OsHd1 has dual effects depending on the photoperiod, as it induces heading under SD conditions but suppresses heading under LD conditions (Ishikawa et al. 2011; Liu et al. 2014; Nemoto et al. 2016). We confirmed that heat stress during grain-filling affects heading date through OsHd1, as HDS plants showed early and delayed heading under SD and LD conditions, respectively, compared with CS plants (Fig. 4c;  Figure S4b). In addition to this early heading phenotype, HDS plants also showed an early diurnal floret opening time compared with CS plants (Fig. 6a to c). Floret opening in many plant species involves the actions of phytohormones including gibberellin, auxin, and ethylene (Ding et al. 2014; Li et al. 2015; Ke et al. 2018; Cheng et al. 2021). JA and its derivatives effectively induce floret opening in rice and other grasses (Wang et al. 2024; Zhu et al. 2024), as defects in JA biosynthesis or signaling lead to delayed floret opening (Ding et al. 2014; Gou et al. 2024). A previous study showed that overexpressing *OsOPR7*, a JA biosynthesis gene, shifts floret opening earlier by 1 h (Wang et al. 2024). Here, heat stress during grain filling also promoted a similar 1-h earlier floret opening in HDS plants through the upregulation of the JA biosynthesis gene *OsAOC1* (Fig. 6e). Early diurnal floret opening has been suggested to help plants achieve pollination before they are exposed to higher temperatures at noon (Che et al. 2024; Wang et al. 2024). Several methods such as genetic modifications or alterations of the genome of early opening *indica* rice cultivars have been proposed to induce early floret opening in rice (Ishimaru et al. 2022; Gou et al. 2024; Zhu et al. 2024). Together with these methods, our study offers an innovative and sustainable method of advancing floret opening in rice without changing the genetic background, by inducing epigenetic changes. As climate change induces high-temperature stress during the growing season of rice, a suitable heading date may help rice plants to escape unfavorable temperatures and other abiotic stresses (Zhou et al. 2021, 2024). In this study, we sowed CS and HDS in May; their derived plants reached the booting stage in the middle of August, which had the highest temperatures throughout the growing season in Japan. Therefore, HDS plants might have developed a heat stress-mitigation mechanism for their reproduction and fitness as they acquired early heading together with early diurnal floret opening compared with CS plants. Indeed, heat stress during grain-filling induced beneficial phenotypic changes in HDS plants; we observed these changes repeatedly in other multiple experimental years under natural conditions (Figure S9a to i).

Heat stress during grain-filling affects transcript levels in the subsequent plants, based on the many DEGs we identified. Yet, the overlap between annotated DEGs in developing plants and seed DMRs was almost not detected. A minimal overlap between DMRs and DEGs has been reported in previous studies, as the position of the DMR located in gene body or the promoter, together with its genomic environment leads to difficulty to predict how a DMR could regulate a gene expression (Havecker et al. 2012; Kawakatsu et al. 2016; Tedeschi et al. 2019). However, when we quantified seed DNA methylation levels at the specific loci at the promoters of target genes, we detected significant differences between CS and HDS plants at the promoter of each target gene (*OsSLB1*, *OsYODA1*, *OsHd1*, and *OsAOC1*) (Fig. 7a to d). The promoter regions of *OsSLB1* and *OsYODA1* promoters were more methylated in HDS than in CS, and later in developing roots (*OsSLB1*) and seedlings (*OsYODA1*) (Fig. 7e and f). Conversely, the promoter regions of *OsHd1* and *OsAOC1* were less methylated in HDS than in CS, as well as in the leaves and lodicules of the subsequent plants (Fig. 7g and h). We propose that this specific maintenance of DNA methylation levels in seeds through to developing organs contributes to the observed differences in transcript levels for each target gene and promotes phenotypic changes in HDS plants. We focused on the role of seed DNA methylation in subsequent development in rice. However, to explain all the changes in transcript levels and other possible traits, for example, stress tolerance, other epigenetic marks should be explored, such as histone modifications (Liu et al. 2019) and post-transcriptional regulation of miRNA (Liu et al. 2021), which are reported to play important roles in transgenerational memory.

In conclusion, we propose that heat stress during grain-filling affects development of the subsequent plants through alterations in seed DNA methylation patterns, which are maintained to some extent within specific organs and likely regulate transcription. Even under nonstress conditions, these phenotypic changes might be one of the adaptive strategies for unpredictable upcoming stresses or environmental changes, which are at the same time profoundly advantageous for raising rice production under anticipated increases in CO_2_ levels and temperatures. In all, these findings suggest a role for seed memory in achieving preferential agronomic traits and acclimation to heat stress caused by climate change.

## Materials and methods

### Plant materials and cultivation methods

To generate control and heat-developed seeds, ten 3-wk-old rice (*Oryza sativa* L. “Nipponbare”) seedlings were transplanted into a 1/5,000-a Wagner pot with 8.75 g of basal dressing fertilizer (N–P_2_O–K_2_O: 4%–4%–4%) and 0.85 g of sigmoid-type coated urea at Kyushu University, Fukuoka, Japan (2016 to 2021 at Hakozaki, 33°67′N 130°42′E; 2019 to 2021 at Ito, 33°37′N 130°25′E) in the middle of June. An additional 0.5 g of ammonium sulfate (N, 21%) was applied once at the tillering stage and again at the panicle booting stage. Plants were grown under natural conditions. Fully developed tillers were removed at the maximum tillering stage, leaving only the main stems to develop. The day when more than 50% of the total population reached anthesis, defined as the day when spikelets on the upper primary rachis branches flowered, with ovule fertilization, was set as the day of flowering (DAF). At 0 DAF, the plants were transferred to 1 of 2 temperature regimes, control (25 °C) or heat (30 °C), in a phytotron with natural light and 70% humidity until harvest at 42 DAF (Figure S1a). Harvested seeds were dried at room temperature for 1 wk and stored at −30 °C to maintain seed vigor before sowing in the following years.

### Global DNA methylation analysis by WGBS

Embryos were carefully dissected from seeds of CS and HDS with a razor blade for embryo isolation. Genomic DNA was extracted from embryos of harvested seeds using an Isospin Plant DNA extraction kit according to the manufacturer's protocol (Nippon Gene). Pooled genomic DNA samples from 200 isolated embryos of each treatment were prepared for WGBS as described (Schmitz et al. 2021). The integrity of the genomic DNA was checked by agarose gel electrophoresis, and the DNA concentration was quantified with Quant-IT PicoGreen (Invitrogen). The WGBS libraries were prepared using an Accel-NGS Methyl-Seq DNA library kit (Swift BioSciences). In brief, 200 ng of genomic DNA was fragmented using a Covaris LE220 focused-ultrasonicator (Covaris, Woburn, MA, USA) to a target fragment size of 400 to 700 bp. The fragmented DNA was then subjected to bisulfite conversion using an EZ DNA Methylation-Gold Kit (Zymo Research), according to the manufacturer's instructions. The resulting bisulfite-treated single-stranded DNA fragments were repaired, and truncated adapters 1 and 2 were ligated to the 3′ and 5′ ends of fragments, respectively. The truncated adapter–ligated DNA was amplified with an indexed primer to complete the WGBS libraries incorporating a full-length adapter. The final libraries were then quantified by qPCR according to the qPCR Quantification Protocol Guide (KAPA Library Quantification kits for Illumina Sequencing platforms), quality-controlled using the TapeStation DNA ScreenTape D1000 system (Agilent), and then sequenced on a HiSeq platform (Illumina, San Diego, CA, USA). For data analysis, after removal of adapter sequences using Trimmomatic v. 0.38 software (Bolger et al. 2014) and of low-quality reads, the clean reads were mapped to the IRGSP-1.0 rice reference genome using Bismark v. 0.22.3 software (Krueger and Andrews 2011) and HISAT2 v2.1.0 (Kim et al. 2019). Gene and TE annotations were obtained from IRGSP-1.0. For control samples, the conversion rate was 56.6% (CpG), 27.0% (CHG), 14.1% (CHH); sequencing depth was 64.3 (without contigs after deduplication); duplication rate was 12.1%; and cytosine coverage for mapping (genome coverage) was 97.9% (without contigs after deduplication). For heat-treated samples, the conversion rate was 57.8% (CpG), 28.5% (CHG), 15.5% (CHH); sequencing depth was 64.9 (without contigs after deduplication), duplication rate was 10.8%, and cytosine coverage for mapping (genome coverage) was 97.9% (without contigs after deduplication). All cytosine reads are summarized in Table S1 and are accessible through the Gene Expression Omnibus (GEO) at NCBI under the accession number GSE203487. Plots of average methylation levels along chromosomes were constructed using Circos software (Krzywinski et al. 2009) with 1-Mb windows. DMRs were defined as 50-bp bins (minimum) containing at least 3 informative cytosines with a methylation delta >0.1 at a significance level of *P* < 0.05 and identified using the DSS v. 2.36.0 package (Wu et al. 2015) in Bioconductor (Gentleman et al. 2004). For DMR distributions, the annotation of DMRs in genes and TEs was performed using HOMER v. 4.11.1 software (Heinz et al. 2010) and the reference IRGSP1.0 rice genome. DMRs within the sequence 3,000 bp upstream or 3,000 bp downstream of annotated protein-coding genes were considered to lie within the promoter region and downstream region, respectively. GO analysis for DMRs was performed using Panther v. 17.0 software (http://pantherdb.org) (Mi et al. 2018), for DMRs within protein-coding genes, using Fisher's exact test with a false discovery rate (FDR) level of *P* < 0.05. The fold enrichment and number of genes involved with each GO term were summarized.

### RNA sequencing and analysis

Total RNA from fully developed leaves from plants derived from CS and HDS and grown under natural conditions at 55 DAS was extracted from frozen materials using the SDS/phenol/LiCl method (Chirgwin et al. 1979). RNA-seq analysis was performed with RNA from 2 biological replicates per treatment. A cDNA library was generated using a NEBNext Ultra II Directional RNA Library Prep Kit (Illumina) and sequenced on an Illumina NovaSeq instrument as 150-bp reads sequencing with reference annotations from IRGSP. Read counts of each gene were calculated using RSEM v. 1.3.0 software (Li and Dewey 2011). Gene expression levels were normalized as counts per million (CPM). DEGs were identified using the edgeR 3.26.3 package (Robinson et al. 2009) in Bioconductor. Genes with *P* < 0.05 (Log_2_FC ≥ 0.37 or Log_2_FC ≤ −0.37) according to the exactTest function of edgeR were considered as DEGs. RNA-seq data can be accessed at NCBI via GEO under the accession number GSE246095. Volcano plots were generated in R v. 4.0.2, and expression heatmaps for tillering- and heading-related genes were generated using fold-change (FC) values from CPM in OriginPro 2021b software (OriginLab). GO analysis for DEGs was performed with Panther v. 17.0 software (http://pantherdb.org) as above for GO analysis, and the number of DEGs was summarized.

### Reverse-transcription quantitative PCR

For RT-qPCR analysis, total RNA was extracted from 3-leaf-stage seedlings, leaves, and roots at the tillering stage (55 DAS), or lodicules (10 AM) using the SDS/phenol/LiCl method (Chirgwin et al. 1979). Total RNA was used as template for reverse transcription using ReverTra Ace reverse transcriptase (Toyobo) according to the manufacturer's instructions. Quantitative PCR was performed on a CFX Connect Optics Module real-time PCR detection system (Bio-Rad) with SYBR Green (Toyobo) as per the manufacturers’ instructions. Primers used for RT-qPCR are listed in the Table S3. All primers used in this study were synthesized by Sigma-Aldrich. PCR thermal cycling conditions were an initial denaturation at 94 °C for 2 min; 40 cycles of denaturation at 94 °C for 20 s, annealing at the primer-specific temperature for 20 s, and extension at 72 °C for 20 s; followed by plate reading. A melting curve was also performed at the end of each run. The results were normalized by the *ddCt* method (Pfaffl 2001) using the expression level of *OsUBQ* (leaf samples) or *OsACTIN* (roots, seedlings, and lodicule samples). Expression heatmaps of genes related to stomatal development and floret opening were plotted using FC values from RT-qPCR data in OriginPro 2021b software (OriginLab).

### Measurement of phenotypes in the subsequent plants

Seeds developed under control conditions (CS) or under heat stress (HDS) were used in this study. The growing regimes are shown in Figure S1b. The meteorological data for precipitations and temperatures during growing season, obtained from Weather station/WEB monitoring system in Kyushu University, are shown in Figure S10. The phenotypes of CS and HDS plants were examined in 2017 to 2024. For the analysis of tiller number, height, and stomatal density, a single 3-wk-old seedling derived from CS or HDS was transplanted into a 1/5,000-a Wagner pot and grown under natural conditions with fertilizer applied as above. Tiller number and plant height measurements were measured. Stomatal density was analyzed by using instant glue on a glass slide to create an imprint of the epidermis as described previously (Kusumi et al. 2012) with adjustments as follows. Imprinted stomata from the middle of a fully expanded leaf from an individual plant during vegetative growth were observed with a ×20 objective lens (BZ-X710, Keyence) and counted using BZ-X Analyzer v. 1.3.1.1 software. Stomata on the abaxial and adaxial surfaces were counted and added to obtain the total stomatal density per mm^2^ unit area. For measurements of heading phenotype and floret opening, ten 3-wk-old seedlings were transplanted into a single 1/5,000-a Wagner pot with the same fertilizer as above. Fully developed tillers were removed at the maximum tillering stage, and only main stems were allowed to develop. For the heading rate, the first day of panicle emergence of each plant was recorded daily at 09:00. A heading histogram was constructed for the total population of plants derived from CS and HDS. The date at which 50% of plants, within a single pot reached heading, was used as the heading date for the entire pot. For floret opening, the florets of headed individual panicles (recorded as the second day of heading), were checked in 1-h intervals during a sunny day. The percentage of opened florets was calculated as the number of open florets at each time point divided by the total number of open florets observed on that day.

### DNA methylation analysis by MeDIP-qPCR

Genomic DNA from isolated embryos of harvested seeds, developing leaves, roots, and lodicules was extracted using a DNeasy Plant Mini Kit (Qiagen) and sheared to about 500 bp by sonication (Picoruptor2, Diagenode). For MeDIP-qPCR, 1,000 ng of sheared DNA was immunoprecipitated with a Methylated DNA Immunoprecipitation Kit (Active Motif) according to the manufacturer's protocol. Immunoprecipitated DNA was subjected to qPCR to identify the locus-specific relative DNA methylation level. The proportion of input was calculated as per the manufacturer's protocol. Specific primers for MeDIP-qPCR are listed in Table S4.

### Yield analysis

For the field experiments, 3-wk-old seedlings derived from CS or HDS were transplanted into the experimental paddy field at Kyushu University with pulverized sigmoid-type basal dressing (N, 41%). Seedlings grown from CS and HDS were arranged in a randomized block design into distinct isolated plots representing 3 replications. Seedlings were transplanted with a 30-cm × 30-cm subplot density. For yield component analysis, plants within 1 m^2^ were harvested. Grain filling and grain weight were calculated from >1.8-mm brown rice. Thousand-grain weight and yield are expressed at 15% grain moisture content. For pot experiments, yield components of a single plant per pot were similarly analyzed as that of field experiment. Harvest index was calculated by dividing total grain yield by biological yield.

### Statistical analysis software

Statistical analyses were performed in SPSS v. 28.0.0.0 software (IBM). Differences among treatments were analyzed by one-tailed Student's *t*-test and Tukey's test. A single plant represents 1 biological replicate in this study.

## Accession numbers

Accession numbers of major genes analyzed in this study are listed in Table S3.

## Supplementary Material

kiag219_Supplementary_Data

## Data Availability

Supporting Information together with materials and methods for this work are available in the Supplementary Information files. Data sets from the WGBS and RNA-seq analyses have been uploaded to the Gene Expression Omnibus (GEO) database, which can be accessed through accession number GSE203487 for WGBS and GSE246095 for RNA-seq.
