# Supplementary material for: Grain-maturing temperature induces seed epi-memory via DNA methylation for subsequent development in rice (Oryza sativa L.)
Source: Plant Physiol. 2026 Apr 21;201(1):kiag219. doi: 10.1093/plphys/kiag219 (PMC13191598; doi:10.1093/plphys/kiag219)
Supplement: kiag219_Supplementary_Data [file kiag219_supplementary_data.pdf]

Grain-maturing temperature induces seed epi-memory via DNA methylation for subsequent development in rice (*Oryza sativa* L.)

Chetphilin Suriyasak[1,2], Yui Oyama[2], Ryusuke Kawaguchi[2], Ryo Matsumoto[2], Yuta Sawada[2], Wun-Jin Chen[2], Hue Thi Nong[2,3], Norimitsu Hamaoka[4], Yushi Ishibashi[1,2]*

[1]Faculty of Agriculture, Kyushu University, Fukuoka, 819-0395, Japan
[2]Graduate School of Bioresource and Bioenvironmental Sciences, Kyushu University, Fukuoka, 819-0395, Japan
[3]Faculty of Biotechnology, Vietnam National University of Agriculture, Hanoi, 131000, Vietnam
[4]Tropical Crop and Environment Section, Institute of Tropical Agriculture, Kyushu University, Fukuoka, 819-0395, Japan
Corresponding author: Yushi Ishibashi
Email: yushi@agr.kyushu-u.ac.jp

Supplemental information
Supplemental Figure S1–S10
Supplemental Table S1–S4

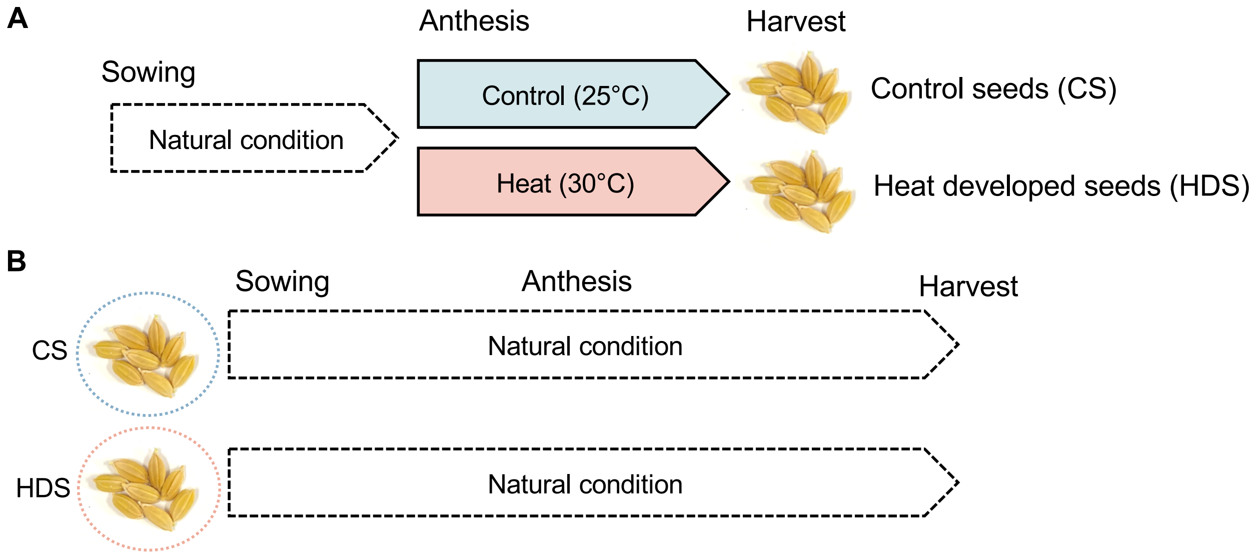

Supplemental Figure S1. Scheme of cultivation and experimental methods used in this study. Cultivation methods for **A)** control seeds (CS) and heat-developed seeds (HDS) and **B)** phenotypic change analysis under natural condition.

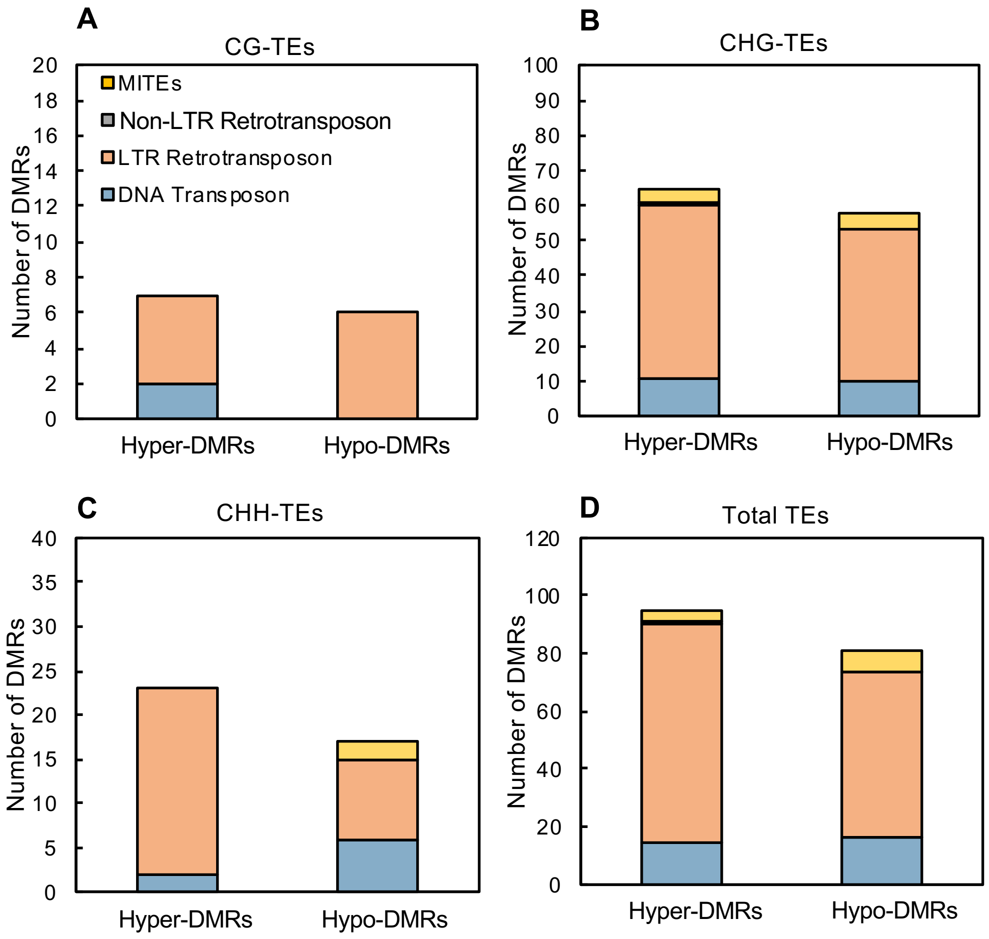

Supplemental Figure S2. Distribution of transposable element (TEs) overlapping with differentially methylated regions (DMRs) of each context. Numbers of DMRs overlapped with **A)** CG-DMRs, **B)** CHG-DMRs, **C)** CHH-DMRs, and **D)** total DMRs (Total hyper-DMRs = 95; total hypo-DMRs = 81).

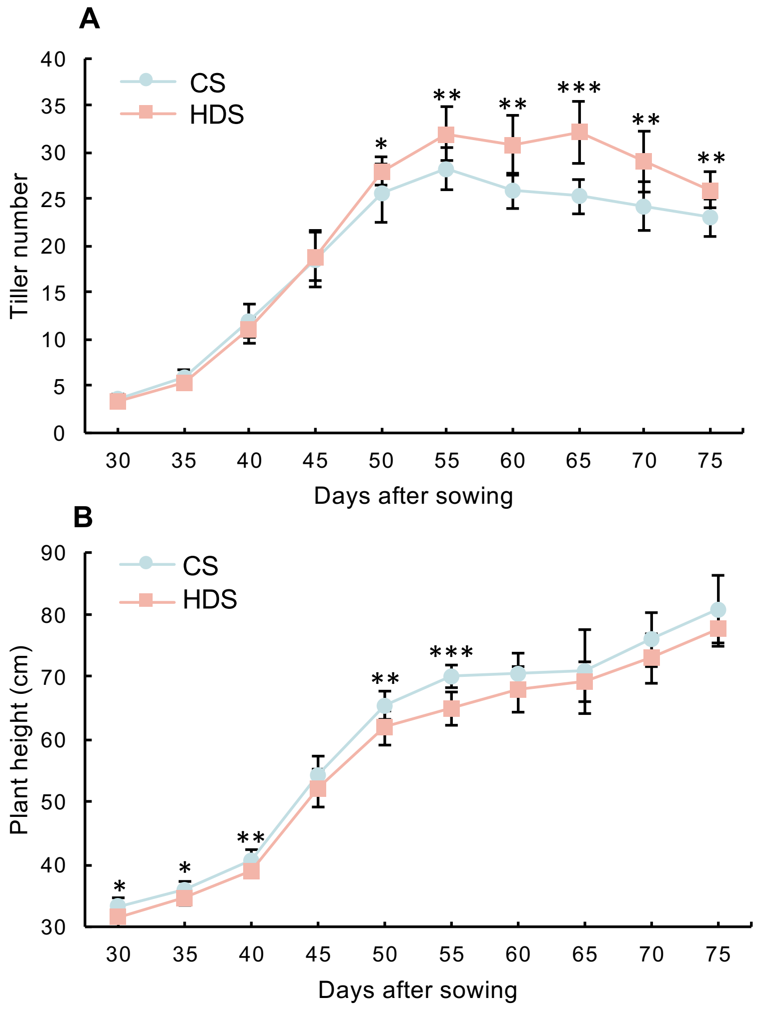

Supplemental Figure S3. Plant height and tiller number during subsequent plant development. A) Tiller number and **B)** plant height of CS and HDS plants after transplantation to heading stage (CS, control seeds; HDS, heat-developed seeds). Significant differences are shown at $P < 0.05$*, $P < 0.01$** and $P < 0.001$*** according to Student's t-test ($n = 8$). Error bars represent SD values.

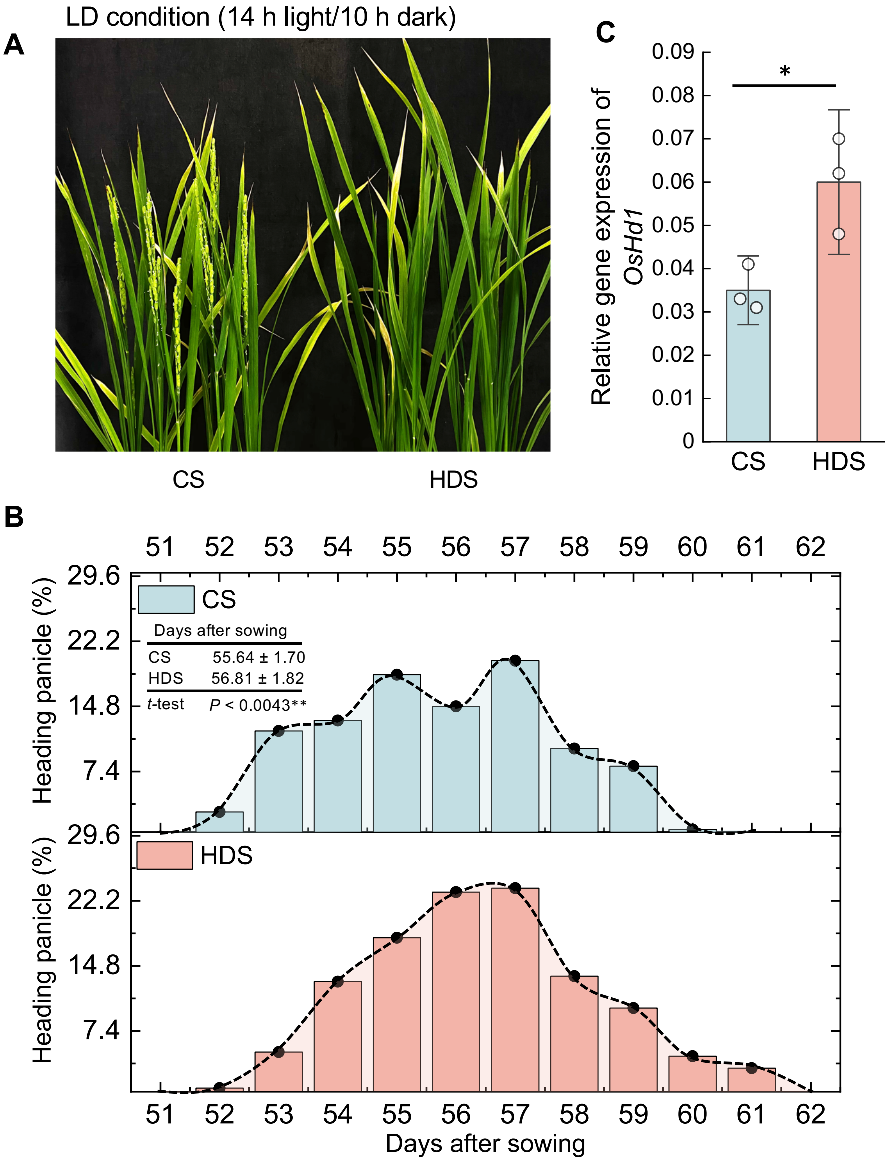

Supplemental Figure S4. Delayed heading of HDS plants with upregulation of *OsHd1* under long-day (LD) condition. A) Heading phenotype and **B)** histograms showing percentages of panicle heading on each date of CS and HDS plants (CS = 250 plants, HDS = 220 plants) with average heading dates ± SD values of the total pots (CS = 25 pots, HDS = 22 pots) are shown at top left (CS, control seeds; HDS, heat-developed seeds). **C)** Relative expression of *OsHd1* in CS and HDS leaves under LD condition (n = 3). Significant differences are shown at P < 0.05* and P < 0.01** according to Student's t-test. Error bars represent SD values.

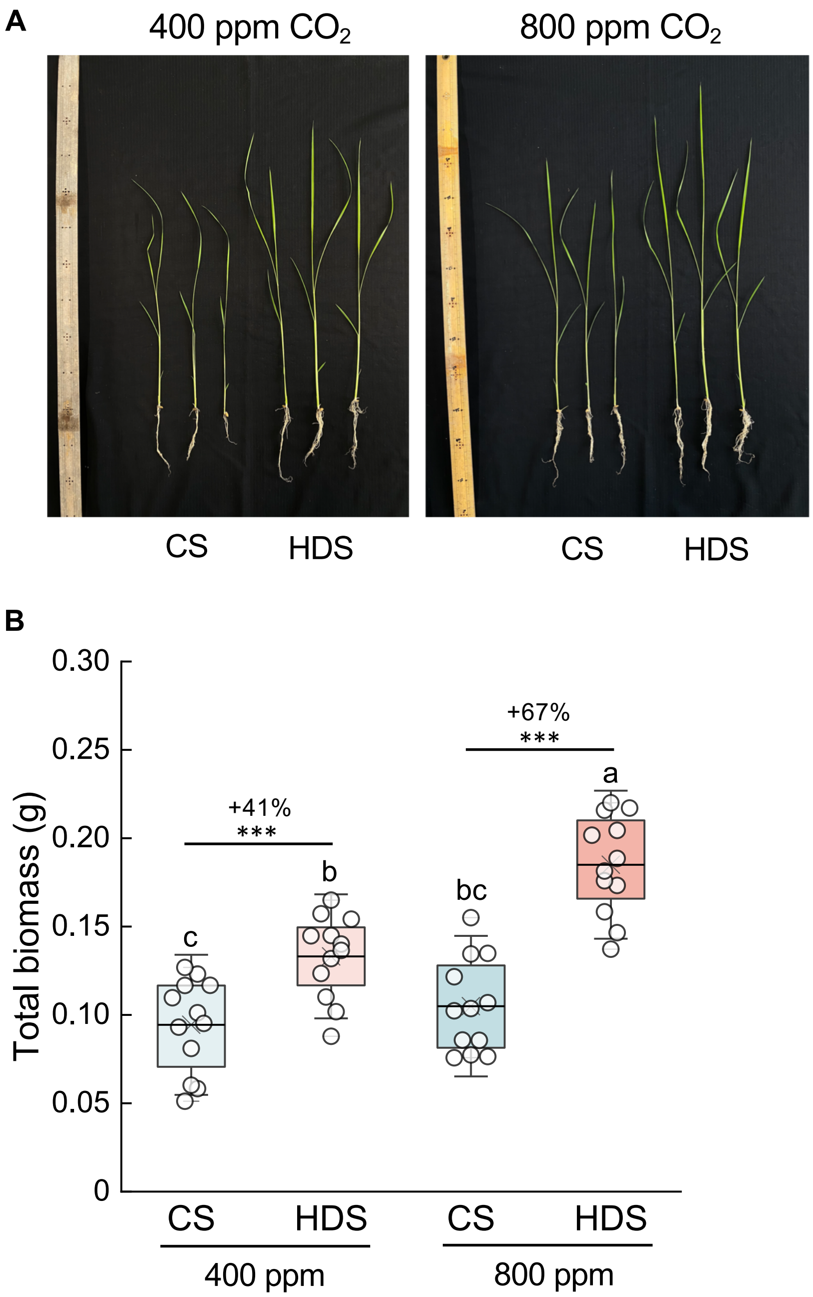

Supplemental Figure S5. Biomass accumulation under ambient and elevated CO_2 conditions of CS and HDS seedlings. A) Plant phenotype and **B)** total biomass of seedlings developed under 400 ppm and 800 ppm CO_2 conditions at 25°C for 3 weeks (CS, control seeds; HDS, heat-developed seeds). Significant differences are shown at P < 0.05* and P < 0.001*** according to Student's *t*-test, and at P < 0.05* by Tukey's test shown as different alphabets (n = 12). In boxplots, the upper and lower bounds represent the 75th and 25th percentiles, respectively. The medians are shown as lines within the box, with the cross marks representing the averages. Whiskers represent SD values.

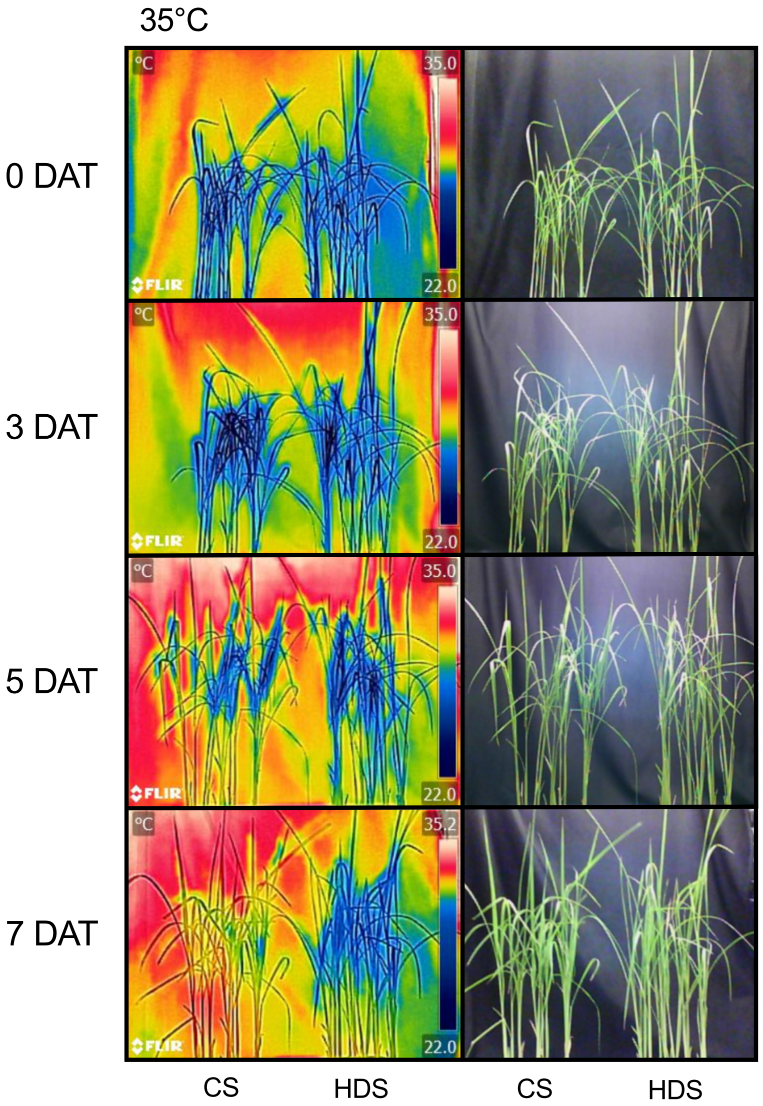

Supplemental Figure S6. Acquired thermotolerance of HDS seedlings. Thermography images of 4-week-old CS and HDS seedlings treated with 35°C heat stress for 7 days (DAT, days after treatment; CS, control seeds; HDS, heat-developed seeds). Color scale bars represent temperature ranges.

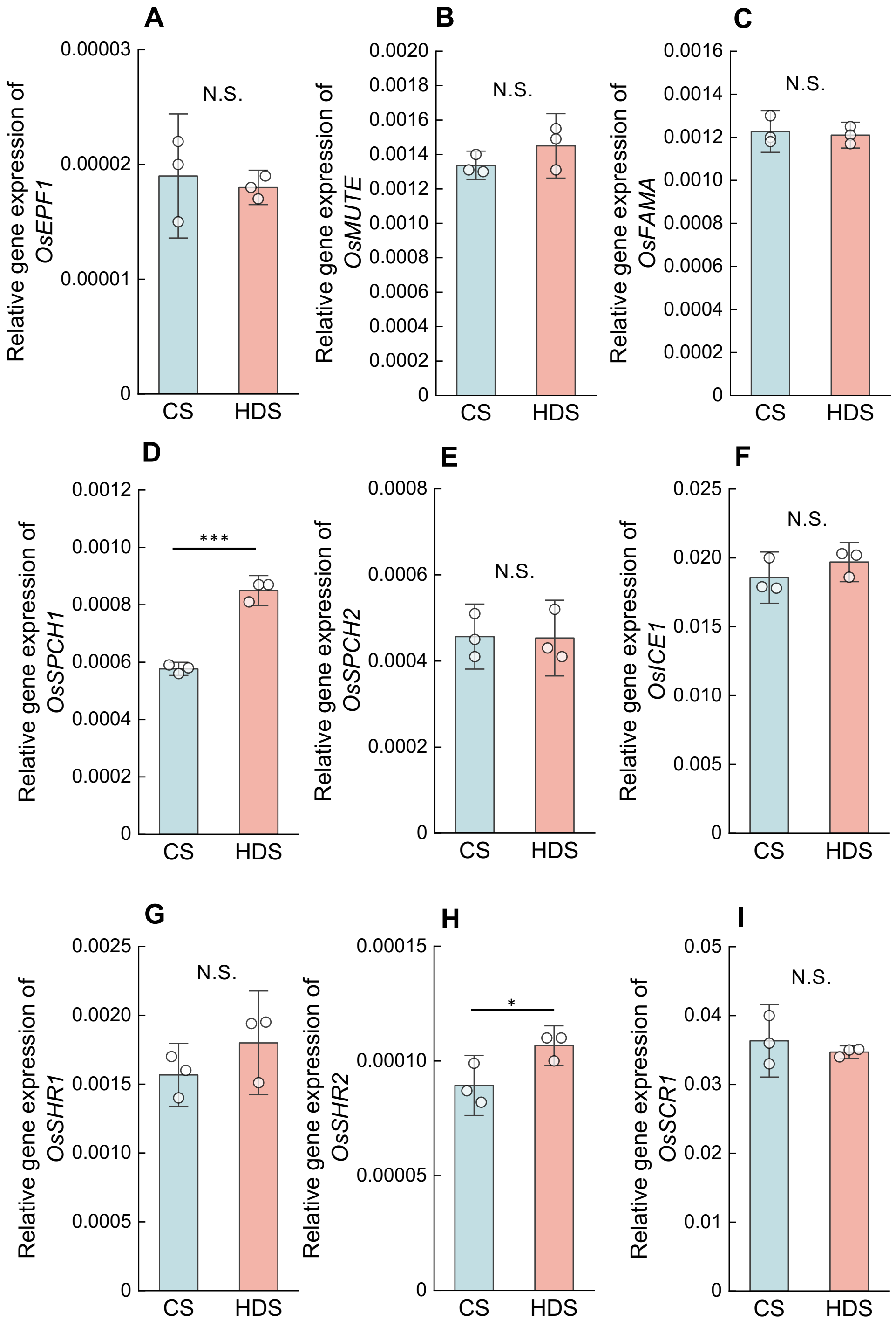

Supplemental Figure S7. Relative expression of stomatal development-related genes. Relative gene expression via RT-qPCR of stomatal development-related genes in CS and HDS developing seedlings (CS, control seeds; HDS, heat-developed seeds). **A)** *OsEPF1*; **B)** *OsMUTE*; **C)** *OsFAMA*; **D)** *OsSPCH1*; **E)** *OsSPCH2*; **F)** *OsICE1*; **G)** *OsSHR1*; **H)** *OsSHR2*; **I)** *OsSCR1*. Significant differences are shown at $P < 0.05$* and $P < 0.001$*** according to Student's *t*-test ($n = 3$), N.S., not significant. Error bars represent SD values.

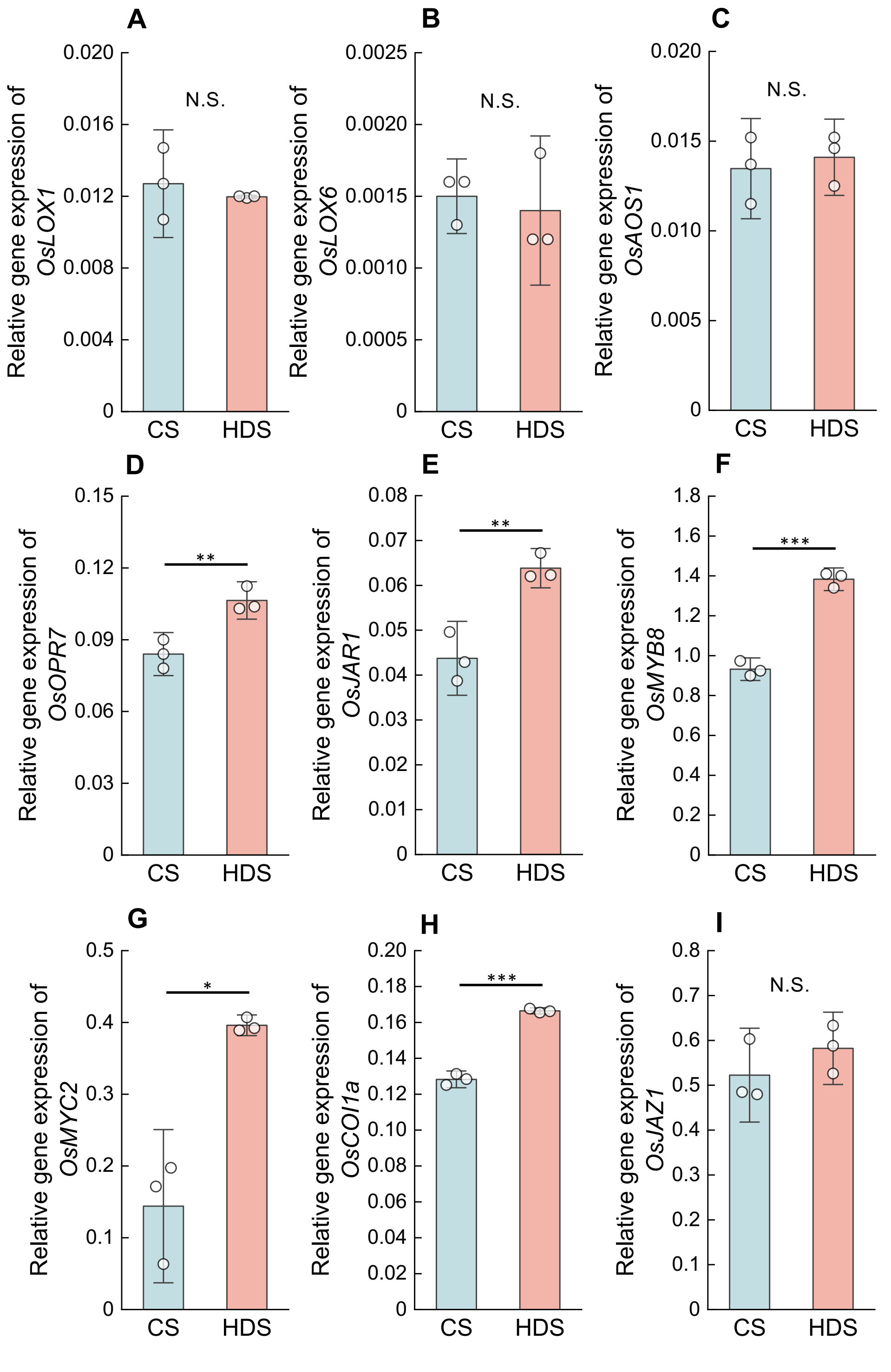

Supplemental Figure S8. Relative expression of floret opening-related genes. Relative gene expression via RT-qPCR of stomatal development-related genes in CS and HDS lodicules at 10:00 AM (CS, control seeds; HDS, heat-developed seeds). **A)** *OsLOX1*; **B)** *OsLOX6*; **C)** *OsAOS1*; **D)** *OsOPR7*; **E)** *OsJAR1*; **F)** *OsMYB8*; **G)** *OsMYC2*; **H)** *OsCOI1a*; **G)** *OsJAZ1*. Significant differences are shown at $P < 0.05$* and $P < 0.001$*** according to Student's *t*-test ($n = 3$), N.S., not significant. Error bars represent SD values.

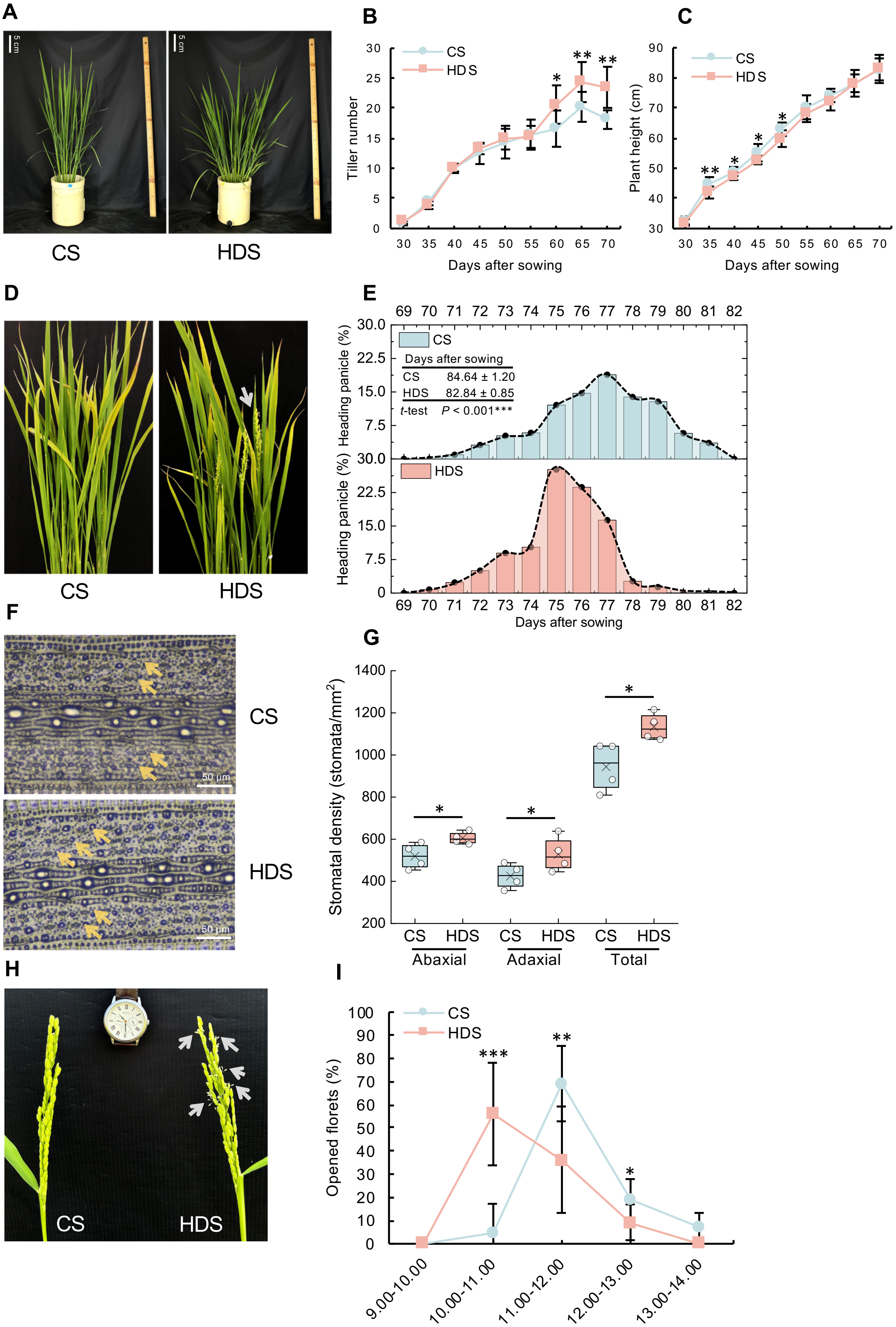

Supplemental Figure S9. Phenotypic changes of HDS plants observed in other replicate years. A) Plant phenotype at maximum tillering stage and **B)**, **C)**, tiller number and plant height of CS and HDS plants in 2023 (n = 8) (CS, control seeds; HDS, heat-developed seeds). **D)** and **E)**, Early heading phenotype of HDS plants and heading histograms showing percentages of panicle heading on each date of CS and HDS plants in 2018 (CS = 1060 plants, HDS = 380 plants), with average heading dates ± S.D. values of the total pots (CS = 106 pots, HDS = 38 pots) are shown at top left. **F)** Imprints of stomata on abaxial surfaces (arrows indicate stomata) and **G)** stomatal density of CS and HDS leaves (n = 4) in 2023. In boxplots, the upper and lower bounds represent the 75th and 25th percentiles, respectively. The medians are shown as lines within the box, with the cross marks representing the averages. Whiskers represent S.D. values. **H)** Plant phenotype (arrows indicate opening florets) and **I)** percentages of opened florets at different time intervals (n = 6). Significant differences are shown at P < 0.05*, P < 0.01** and P < 0.001*** according to Student's t-test. Error bars represent SD values.

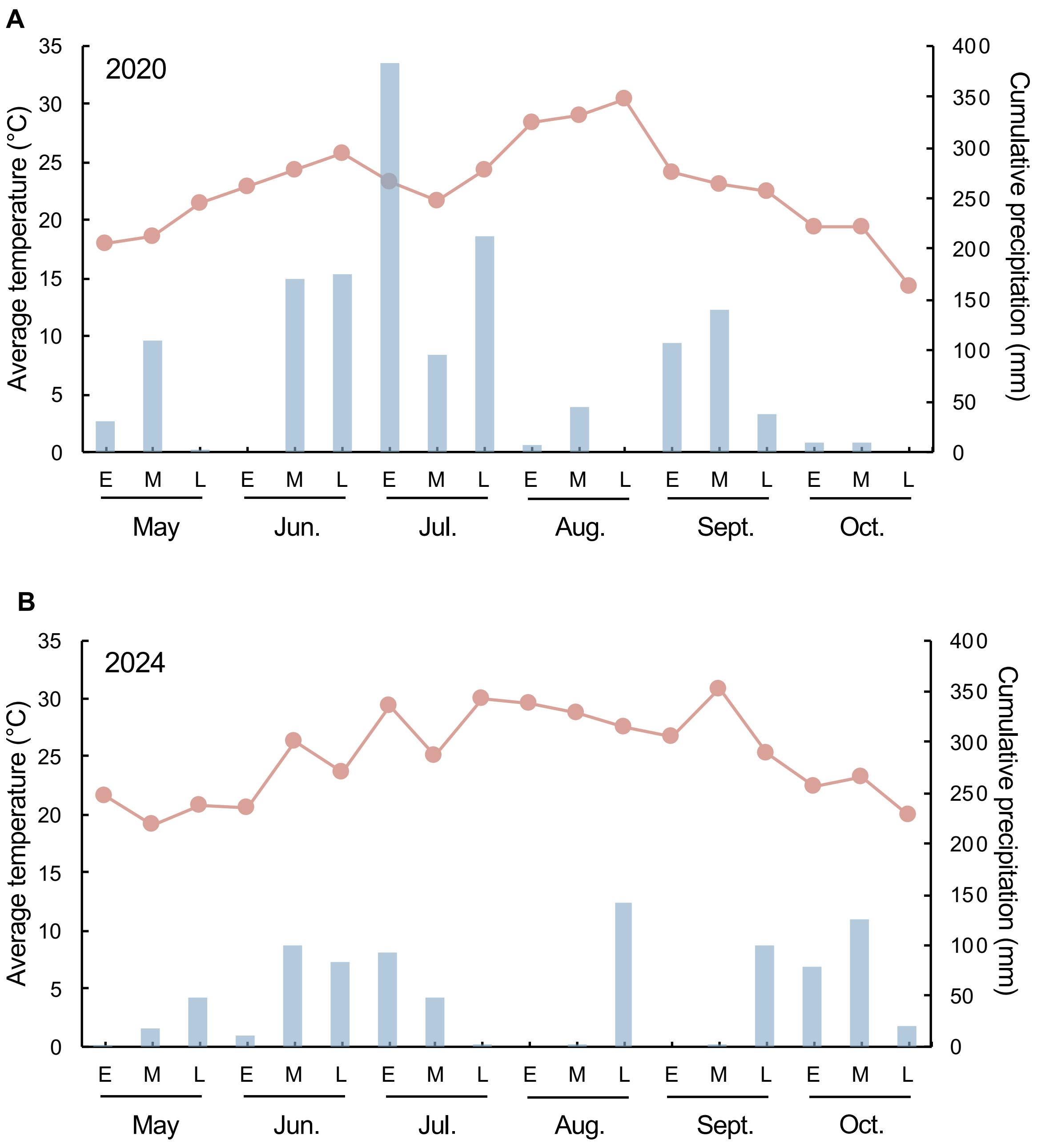

Supplemental Figure S10. The meteorological data for precipitations and temperatures during growing seasons of field cultivations. Average temperatures and cumulative precipitations during field cultivations in **A)** 2020 and **B)** 2024. E, early; M, middle; L, late; period of each month. Red lines and blue bars represent average temperatures (left y-axis) and cumulative precipitations (right y-axis), respectively. Data were obtained from The meteorological data for precipitations and temperatures during growing season, obtained from Weather station/WEB monitoring system in Kyushu University, Ito campus (Ito, 33°37′N 130°25′E).

Supplemental Table S1. Total cytosines in DNA from control seeds (CS) and heat-developed seeds (HDS) analyzed by whole-genome bisulfite sequencing

	CS	HDS
Total cytosines analysed	3,919,488,890	3,991,072,994
Duplication rate (%)	12.1%	10.8%
Coverage cytosine for mapping (%)	97.9%	97.9%
Methylated cytosines in CpG context	356,642,852	369,500,795
Methylated cytosines in CHG context	166,128,581	177,195,999
Methylated cytosines in CHH context	375,821,081	423,437,019
Unmethylated cytosines in CpG context	273,533,402	270,050,603
Unmethylated cytosines in CHG context	449,808,489	443,743,999
Unmethylated cytosines in CHH context	2,297,554,485	2,307,144,579
CG methylation (%)	56.6%	57.8%
CHG methylation (%)	27.0%	28.5%
CHH methylation (%)	14.1%	15.5%

Supplemental Table S2. Yield and yield components of control seeds (CS) and heat-developed seeds (HDS) in pot experiments in two replicate years in 2019 and 2023 under natural conditions.

**Pot experiment (2019)**	**Panicle number**	**Spikelet/ panicle**	**Grain filling (%)**	**1000-grain weight (g)**	**Total yield (g/pot)**
CS	19.25 ± 1.48	67.76 ± 4.42	88.68 ± 3.67	25.05 ± 1.41	28.25 ± 2.11
HDS	21.23 ± 2.13	67.07 ± 5.32	88.73 ± 2.71	25.25 ± 1.33	32.34 ± 2.49
P-value (n = 8)	***P* = 0.012***	*P* = 0.392	*P* = 0.464	*P* = 0.389	***P* = 0.005****

**Pot experiment (2023)**	**Panicle number**	**Spikelet/ panicle**	**Grain filling (%)**	**1000-grain weight (g)**	**Total yield (g/pot)**
CS	16.12 ± 1.95	82.30 ± 11.41	87.51 ± 6.25	20.40 ± 1.01	23.78 ± 5.53
HDS	21 ± 2.82	82.39 ± 10.55	91.73 ± 3.52	20.95 ± 0.29	32.99 ± 4.00
P-value (n = 8)	***P* = 0.0008*****	*P* = 0.43	*P* = 0.062	*P* = 0.087	***P* = 0.0011****

Values are means ± SD (n = 3). Asterisks indicate significant differences, as determined by a Student's *t*-test (*, $P < 0.05$). CS, control seeds; HDS, heat-developed seeds.

Supplemental Table S3. RT-qPCR primers used in this study

Gene	RAP ID	Forward	Reverse
OsActin	Os11g0163100	GACTCTGGTGATGGTGTCAGC	GGCTGGAAGAGGACCTCAGG
OsUBQ	Os03g0234200	AACCAGCTGAGGCCCAAGA	ACGATTGATTTAACCAGTCCATGA
OsSLB1	Os01g0700900	CAGGTTCTCAAGAGGCTTCG	GGAGGTGCTCGTAAGTGAGC
OsHd1	Os06g0275000	TCAGCAACAGCATATCTTTCTCATCA	TCTGGAATTTGGCATATCTATCACC
OsHd3a	Os06g0157700	GCTCACTATCATCATCCAGCATG	CCTTGCTCAGCTATTTAATTGCATAA
OsYODA1	Os04g0559800	CTGCAATGTTCAAGATCGGAAA	TAACAGCCAAATGTTCCAATGG
OsMUTE	Os05g0597000	CCGTCAGGATCATCGCCCTT	CTGGTGGACCTCCATGGCAA
OsFAMA	Os05g0586300	CAGCAGAGAAGCCACCTGGA	AGCACGTCGGAGAAGGTGAG
OsSPCH1	Os06g0526100	GCAACCGGAGGAAGCAGATG	CTTCTTGGCCTCCAGCGAGT
OsSPCH2	Os02g0257500	AACCTGGTGCTGAAGACGGT	ACTCAGCTCGCACTCGATCC
OsICE1	Os11g0523700	CGCATGGGTCCATGATGCTG	GAGATGCTGGTCGTGGTGGT
OsSHR1	Os07g0586900	GTACCAAGAAGAATGCGGCAAC	AGCTCGTTGAGCATCCACATG
OsSHR2	Os03g0433200	ACCCTCTTCAGGTTGGTTAGCC	GTGGAACTGGCGGGAGGAAG
OsSCR1	Os11g0124300	CTTCCGTGTGTCGTCGCTTG	AGCAGAGGCAGTGAGAAGGC
OsEPF1	Os04g0457700	CCCACTGGAGTACGTACTAATC	ATAATGAACGAAATGTGGGCAC
OsLOX1	Os03g0700700	CAGCAAGATCACCGAAGCTC	AGAATGGTCCTGCTCGCATA
OsLOX6	Os04g0447100	TTCGCCTACCGGGAACTATG	TGCCTCTACACGACACAACC
OsAOS1	Os03g0767000	CAGCAGGTGATCCCCAAGTT	CTGGCAGAGGAAGCCGAAC
OsAOC1	Os03g0438100	GCTACGAGGCCATCTACAGC	TGCCCTTGAGGTAGAAGGTG
OsOPR7	Os08g0459600	CATTGAGATCCATGGTGCTC	CTGAATCATAGGCGTCAAGG
OsJAR1	Os05g0586200	GAGCTTCGTGGAACGATCAA	TGTAGCCTGCGTCAATGAAG
OsMYB8	Os01g0637800	GTCTGGGATGAGTACATGCA	GTCCACGAAGTTCCAGAAG
OMYC2	Os10g0575000	TGGACGTGTACCATGCCAG	TTGAGCTGGTCCTGCGAGTA
OsCOI1a	Os01g0853400	CCAATGCGCTGCAAGACTTT	TCATACTTGGTGAGCTCTCCTACCT
OsJAZ1	Os04g0653000	CAGCAGGTTGGTGAGCAAAG	TCCATCCCTGATGCTTCCAT

Stomatal development and JA-related primers were obtained from Shimizu *et al*. (2013), Ding *et al.* (2014), Verma *et al.* (2020), Li *et al*. (2021), He *et al.* (2021) and Gou *et al.* (2024).

References

T. Shimizu *et al*., *OsJAR1* contributes mainly to biosynthesis of the stress-induced jasmonyl-isoleucine involved in defense response in rice. *Biosci. Biotechnol. Biochem.* **77**, 1556–2013 (2013).
W. Ding *et al*., A jasmonate-mediated regulatory network modulates diurnal floret opening time in rice. *New Phytol.* **244**, 176–191 (2014).
R.K. Verma *et al*., Overexpression of Arabidopsis ICE1 enhances yield and multiple abiotic stress tolerance in indica rice. *Plant Signal. Behav*. **15**, 1814547 (2020).
Y. He *et al*., Jasmonic acid plays a pivotal role in pollen development and fertility regulation in different types of P(T)GMS rice lines. *Int. Mol. Sci.* **22**, 7926 (2021).
Z. Li *et al*., *OsBC1L1* and *OsBC1L8* function in stomatal development in rice. *BBRC* **576**, 40–47 (2021).
Y. Gou *et al*. Natural variation in *OsMYB8* confers diurnal floret opening time divergence between indica and japonica subspecies. *Nat. Commun.* **15**, 2262 (2024).

Supplemental Table S4. MeDIP-qPCR primers used in this study

Gene	Forward	Reverse
OsSLB1 promoter P1	TCATTCACAACACACTTGAAGCTTGA	TCCGACTTCCTCTACTCCACCTCT
OsSLB1 promoter P2	ACGGTGTAGACATGGTGTTTGGGT	ATGGCTCTACAGCGTAACCGGGTA
OsSLB1 promoter P3	TACCCGGTTACGCTGTAGAGCCAT	TGTCAGCACGGAAGATACCGATGA
OsYODA1 promoter P1	CAAAAGATGGTGGCTAGATGATCTGGGT	AAGTACTACACTTTCACTCGCTACAC
OsYODA1 promoter P2	TCGGTTTAATTGTACACCGAATCACC	TGCCATCAGCATGCCTATCGTTAC
OsYODA1 promoter P3	AACGGACCGTCTCGATGAAGCTAC	GCGCATCGGGGTAGCAACGGTTC
OsHd1 promoter P1	ATGGCTCTACAGCGTAACCGGGTA	CGTCGCCGATTTCAATTCGAAAAT
OsHd1 promoter P2	GTACATCATCACAGTGGCTTCCAATTCT	AAGTATCTAACCTGAACTGATGTCAATACC
OsHd1 promoter P3	TGTCAATCGCTGGATTCGACTTGA	GAACGAAGTAGCCTTGCTTGTGGT
OsAOC1 promoter P1	TGTCTACAAATCTGTAGCCCGTTGC	CCAGGTTTTGCATGAGAGATAGTTT
OsAOC1 promoter P2	AAACTATCTCTCATGCAAAACCTGG	AAGTGAGCAGTGATAAGGCCAGTCC
OsAOC1 promoter P3	GGACTGGCCTTATCACTGCTCACTT	TGCCATTTGGAGGAACTAAACAGGTC
